# Impacts of dietary sodium alginate as a prebiotic on the oriental river prawn (*Macrobrachium nipponense*): A comprehensive analysis of growth, physiology, immunity, antioxidant, and metabolism

**DOI:** 10.1016/j.vas.2025.100516

**Published:** 2025-09-30

**Authors:** Mohammad Ettefaghdoost, Hossein Haghighi

**Affiliations:** Fisheries Department, Faculty of Natural Resources, University of Guilan, Sowmeh Sara, Guilan, Iran

**Keywords:** Growth, Metabolism, Polysaccharide, Sodium alginate, *Macrobrachium nipponense*

## Abstract

The effects of dietary sodium alginate supplementation on *Macrobrachium nipponense* were evaluated over a 60-day feeding trial, with emphasis on growth, hematological, immune-physiological responses, and metabolic characteristics. Juvenile prawns with an initial average weight of 1.47 ± 0.05 g were assigned to diets containing 0.0 (control), 0.5, 1.0, 2.0, or 4.0 g/kg sodium alginate. Among all the examined water quality indicators, dissolved oxygen (DO) was the only parameter that showed a statistically significant rise with increasing levels of sodium alginate supplementation, with the 4.0 g/kg group showing the maximum value of DO concentration (*P* < 0.05). Furthermore, growth metrics and survival rates (SR) were significantly enhanced in prawns receiving 2.0 and 4.0 g/kg sodium alginate compared to other treatments (*P* < 0.05). Hemato-biochemical indices generally decreased following sodium alginate supplementation, whereas levels of high-density lipoprotein (HDL) and low-density lipoprotein (LDL) were elevated (*P* < 0.05). Most immune parameters and antioxidant enzyme activities demonstrated positive modulation in response to the dietary intervention (*P* < 0.05), although glutathione peroxidase (GPx), acid phosphatase (ACP), and alkaline phosphatase (AKP) activities did not differ significantly among groups (*P* > 0.05). Diets supplemented with 4.0 g/kg sodium alginate notably enhanced digestive enzyme activities, promoted beneficial intestinal microbiota, and improved body composition (*P* < 0.05). Additionally, sodium alginate intake influenced the expression of genes associated with growth, immune function, and metabolism (*P* < 0.05). Overall, these findings support the dietary inclusion of 4.0 g/kg sodium alginate to optimize growth, physiological condition, and metabolic health in *M. nipponense*.

## Highlights

• Sodium alginate improved growth and feed efficiency in *M. nipponense*.
• Digestive enzyme activity significantly increased under sodium alginate-supplemented diets.
• Antioxidant defenses enhanced via reduced MDA and elevated T-AOC levels.
• Gut microflora shifted toward beneficial LAB in response to sodium alginate supplementation.
• Gene expression of markers related to growth, immunity, and metabolism was upregulated by sodium alginate.

## Graphical abstract

**Figure:**
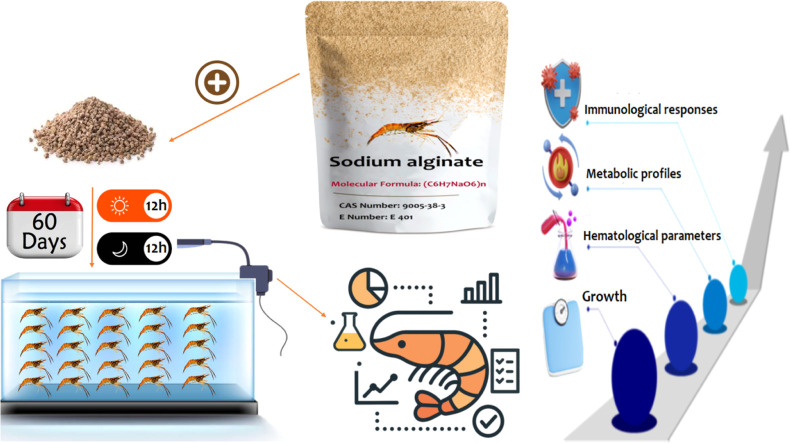

## 1 Introduction

Aquaculture has emerged as one of the fastest-growing food-producing sectors worldwide, playing a pivotal role in global food security and nutrition (Naylor et al., 2021; Bu et al., 2024). The rapid intensification of aquaculture systems has introduced a myriad of challenges, including disease outbreaks, environmental stress, and compromised growth performance (Defoirdt et al., 2011; Alvanou et al., 2023; Bondad‐Reantaso et al., 2023). These issues necessitate the development and application of functional feed additives that can enhance the health, immunity, and overall performance of cultured species (Ma & Hu, 2024; Onomu & Okuthe, 2024; Rocha et al., 2024). Among the various strategies to improve aquaculture productivity and sustainability, the use of natural bioactive compounds has garnered significant attention (Niculescu & Grumezescu, 2022; Ma & Hu, 2024; Ettefaghdoost et al., 2025a). In particular, polysaccharides derived from natural sources such as algae, fungi, and plants have shown promise as functional feed additives (Vidhya Hindu et al., 2019; Goh et al., 2023; Liu et al., 2025). These biopolymers exhibit diverse biological properties, including immunomodulatory, antioxidative, and antimicrobial effects, which are beneficial for aquatic animals (Ma & Hu, 2024; Onomu & Okuthe, 2024; Liu et al., 2025).

One such polysaccharide is sodium alginate, a salt of alginic acid extracted primarily from brown seaweeds (Phaeophyceae). Sodium alginate has been extensively studied in the biomedical and food industries for its biocompatibility, gelling properties, and stability (Hecht & Srebnik, 2016; Sanchez-Ballester et al., 2021; Wang et al., 2025). In aquaculture, sodium alginate is gaining recognition as a potential dietary supplement due to its positive effects on growth performance, immune response, gut health, and stress resistance in various aquatic species (Cheng et al., 2005; Abbas et al., 2023; Bai et al., 2023; Kong et al., 2025; Saleh et al., 2025). Sodium alginate is composed of linear chains of β-d-mannuronic acid (M) and α-l-guluronic acid (G), forming blocks of M-M, G-G, or alternating M-G residues. These structural characteristics contribute to its high viscosity, water-binding capacity, and bioactivity (Abbas et al., 2023; Bai et al., 2023). When incorporated into aquafeeds, sodium alginate can modulate the intestinal microbiota, stimulate the innate immune system, and improve nutrient absorption efficiency (Cheng et al., 2005; Kong et al., 2025; Saleh et al., 2025). Studies have demonstrated that sodium alginate supplementation enhances lysozyme activity, respiratory burst, and phagocytic activity in fish and crustaceans (Abbas et al., 2023; Bai et al., 2023; Saleh et al., 2025). Additionally, sodium alginate can serve as a prebiotic, promoting the proliferation of beneficial gut bacteria while suppressing pathogenic strains, thereby contributing to better gut health and disease resistance (Kong et al., 2025; Saleh et al., 2025). These attributes make sodium alginate a promising candidate for enhancing the physiological resilience and performance of farmed aquatic animals (Cheng et al., 2005; Abbas et al., 2023; Bai et al., 2023; Kong et al., 2025; Saleh et al., 2025). Several previous nutritional investigations have explored the application of sodium alginate in crustacean aquaculture. For instance, Cheng et al. (2005), Santos et al. (2019) and Bai et al. (2023) demonstrated that dietary sodium alginate improved growth performance and modulated immunological responses in Pacific white shrimp (*Litopenaeus vannamei*), contributing to enhanced disease resistance. In another relevant study, Liu et al. (2006) investigated the effects of sodium alginate-based diets in giant tiger prawn (*Penaeus monodon*), observing elevated stress tolerance, feed efficiency, and survival rates under intensive farming conditions. These findings collectively support the broader applicability of sodium alginate as a functional feed additive in crustacean culture.

*Macrobrachium nipponense*, commonly known as the oriental river prawn, is a freshwater crustacean species that is already commercially cultured in several East Asian countries (China, Japan, and South Korea), where it is valued for its rapid growth, adaptability, and strong market demand (New, 2005; De Grave & Ghane, 2006; Afanasyev et al., 2020). It is particularly valued in countries such as China, Japan, and Korea for its fast growth, adaptability to diverse environmental conditions, and high market demand (New, 2005; New & Nair, 2012; Marques et al., 2016; Luo et al., 2025). Due to its favorable characteristics, including high reproductive potential, good feed conversion efficiency, and resistance to certain diseases, *M. nipponense* has become a preferred candidate for freshwater prawn aquaculture (Jian et al., 2024; Zhou et al., 2024; Ettefaghdoost & Haghighi, 2025; Ettefaghdoost et al., 2025b). Moreover, its ecological compatibility and ability to coexist with other aquaculture species in polyculture systems make it an attractive option for integrated farming practices (New, 2005; New & Nair, 2012; Marques et al., 2016; Afanasyev et al., 2020). Despite its economic significance, the intensification of *M. nipponense* farming has led to concerns regarding health management, disease outbreaks, and fluctuating growth performance, emphasizing the need for effective dietary interventions (New, 2005; New & Nair, 2012; Luo et al., 2025).

This study examines the functional potential of sodium alginate in the culture of *M. nipponense* in response to the growing demand for sustainable and antibiotic-free strategies in aquaculture. With established immunostimulatory and prebiotic properties, sodium alginate is hypothesized to modulate growth, oxidative balance, and non-specific immunity. By addressing existing knowledge gaps, the findings are expected to inform the development of eco-friendly and cost-effective feed formulations for freshwater prawns and other commercially relevant crustaceans.

## 2 Materials and methods

### 2.1 Prawn rearing and experimental setup

The 60-day experimental trial was carried out at the Sadaf-Aquarium Culture Facility (Rasht, Gilan, Iran). Specimens of freshwater prawn were collected (Filmar, Large Holding Trap Net, Quebec, Canada) from the Hend Khaleh River, located in Gilan Province, Iran (coordinates: 37°22′55″N, 49°26′39″E). Individuals selected for the study weighed (A&D, GF-20 K, Tokyo, Japan) between 1.0 and 1.5 *g* and measured (Mitutoyo, 50,0-196-30, Kawasaki, Japan) 5.0 to 5.5 centimeters in length. Prior to the trial, prawns were acclimated for two weeks in a 1000-liter holding tank (Polymaster, FT1000B, Victoria, Australia) and provided with a standardized diet containing 45–46 % protein, 5–6 % lipid, 13–14 % ash, 9–10 % moisture, and an energy value of 18 kJ/g, with a particle diameter of 1 mm (Ettefaghdoost et al., 2018). Following acclimation, biometric assessments were conducted using a precision electronic scale (A&D, GF-20 K, Tokyo, Japan) and caliper (Mitutoyo, 50,0-196-30, Kawasaki, Japan). A completely randomized design was employed to assign prawns into five dietary treatment groups, each with three replicates (15 tanks total), stocking 25 prawns per tank (13♂:12♀). Initial mean body weight and length were 1.47 ± 0.05 g and 5.41 ± 0.07 cm, respectively. Each experimental unit (aquarium) had a 100-liter capacity filled with dechlorinated tap water. One-third of the water volume was exchanged daily, with full water replacement conducted during bioassay procedures. Water dechlorination was achieved by continuous aeration for 24 h prior to use. Aeration in all tanks was maintained via a centralized air supply system (Pentair, PT620, Apopka, USA). The photoperiod was regulated to 12 h light and 12 h darkness using LED illumination (GreenSun, Submersible LED Bar, Beijing, China).

### 2.2 Preparation of experimental diets

Experimental diets were formulated using a nutrient model specifically optimized for *Macrobrachium* spp*.*, with all ingredients ground (Pulverisette, Pulv-14, Idar-Oberstein, Germany), sieved (BIOBASE, BK-TS200, Shandong, China), weighed (Shimadzu, UP4201Y, Kyoto, Japan), and mixed (Turbula, T2C, Muttenz, Switzerland) thoroughly to form a uniform paste, which was extruded into strands of 1 mm diameter (APV, MicroLab Twin-Screw, Crawley, United Kingdom) (Ettefaghdoost & Haghighi, 2021). In the final formulation stage, sodium alginate (Sigma-Aldrich®, Product No. W201502, CAS No. 9005–38–3, MDL No. MFCD00081310, E Number: E 401, St. Louis, USA) was dissolved in double distilled water (Zolal Teb, Catalogue No. DE-20/1, Tehran, Iran) using a magnetic stirrer (iGene Labserve, IGN PRO, Mumbai, India) and then uniformly sprayed (Bürkle, LaboPlast 500 mL, Grossostheim, Germany) onto the diets. The feeds were then dried (Memmert, ULE 500, Schwabach, Germany) and stored at −18 °C (Labcold, UF-18, Derbyshire, UK), with daily feeding portions kept at 4 °C (Labcold, LBB12, Derbyshire, UK) prior to administration. Five dietary treatments were developed containing sodium alginate at inclusion levels of 0.0 (control), 0.5, 1.0, 2.0, and 4.0 g/kg. Prawns were hand-fed to apparent satiation at three intervals daily (08:00, 14:00, and 20:00). The feed quantity per meal was measured with a digital scale (Ohaus, Pocket Pro 200, accuracy: 0.01 g, Parsippany, USA) before each feeding session. Feed allowance was adjusted daily based on consumption patterns to prevent residual feed, which was manually removed (PYREX, Corning Inc., 1000-L, New York, USA) two hours post-feeding, dried at 70 °C, and weighed. The ingredient composition of the experimental diets is presented in Table 1.

**Table 1:** Ingredients and nutritional composition of the experimental diets employed in the current study (%, dry matter basis).

Sodium alginate (g/kg)					
	0.0	0.5	1.0	2.0	4.0
Ingredients (%)					
Fish meal^1^	30.00	30.00	30.00	30.00	30.00
Soy meal	30.00	30.00	30.00	30.00	30.00
Wheat meal	7.00	7.00	7.00	7.00	7.00
Corn meal	7.00	7.00	7.00	7.00	7.00
Casein^2^	16.00	16.00	16.00	16.00	16.00
Vitamin premix^3^	2.00	2.00	2.00	2.00	2.00
Mineral premix^4^	2.00	2.00	2.00	2.00	2.00
Cholesterol^5^	0.20	0.20	0.20	0.20	0.20
Vitamin C^6^	0.10	0.10	0.10	0.10	0.10
Dicalcium phosphate^7^	0.50	0.50	0.50	0.50	0.50
Filler (CMC) premix^8^	5.20	5.15	5.10	5.00	4.80
Sodium alginate^9^	0.00	0.05	0.10	0.20	0.40
Nutritional composition					
Moisture (%)	9.65	9.77	9.59	9.81	9.70
Crude protein (%)	44.91	45.11	44.96	45.08	45.02
Crude lipid (%)	5.01	5.13	5.06	5.09	5.03
Fiber (%)	2.95	3.08	2.98	3.04	2.91
Ash (%)	12.96	13.04	12.89	13.12	12.96
Nitrogen-free extract (%)	24.52	23.87	24.52	23.86	24.38
Gross energy (kJ/g)^10^	19.67	19.72	19.88	19.55	19.97

1 Arad-Powder® (Tehran, Iran).

2 Quelab Inc. (CAS No. 9000–71–9, EC No. 232–555–1, Montréal, Canada).

3 Aras-pharmaceutical Co. (Aquavit-1®, Tehran, Iran) – Each 1000 g of the vitamin premix comprised; Vitamin A (retinol 1200,000 IU), Vitamin B_1_ (thiamin 2500 mg), Vitamin B_2_ (riboflavin 4000 mg), Vitamin B_6_ (pyridoxine 2500 mg), Vitamin B_7_ (Biotin 150 mg) Vitamin B_9_ (folate 1000 mg), Vitamin B_12_ (cobalamin 8 mg), Vitamin C (ascorbic acid 30,000 mg), Calcium pantothenate (10,000 mg), Vitamin D_3_ (cholecalciferol 400.000 IU), Vitamin K_3_ (menadione 800 mg), Niacin (nicotinic acid 35,000 mg).

4 Sci Laboratories Inc. (Qazvin, Iran) – Each 1000 g of the mineral premix comprised; Cobalt (Co 100 mg), Copper (Cu 600 mg), Choline chloride (6000 mg), Iodine (I 600 mg), Iron (Fe 6000 mg), Manganese (Mn 5000 mg), Selenium (Se 20 mg), Zinc (Zn 10,000 mg).

5 Merck Group (CAS No. 57–88–5, EC No. 200–353–2, Darmstadt, Germany).

6 Aras-pharmaceutical Co. (Aquavit-C®, Tehran, Iran) – Each 500 g Vitamin C premix contained; Stay-C 35 %.

7 Aras-pharmaceutical Co. (CAS No. 7757–93–9, EC No. 231–826–1, Tehran, Iran).

8 Kimia-Lab Inc. (Tehran, Iran).

9 Sigma-Aldrich®, Product No. W201502, CAS No. 9005–38–3, MDL No. MFCD00081310, E Number: E 401, St. Louis, USA).

10 The energy values for protein, lipid, and carbohydrate are quantified as 16.7 kJ/g, 37.6 kJ/g, and 16.7 kJ/g, respectively.

### 2.3 Water quality monitoring

Water quality parameters were monitored following the methodological framework outlined by APHA (2012). Dissolved oxygen concentrations were recorded using a calibrated oxygen meter (YSI, ProODO, Yellow Springs, USA), while water temperature was measured with a waterproof digital thermometer (Hanna Instruments, HI98509, Woonsocket, USA). Additional key indicators—including nitrate, total dissolved solids (TDS), nitrite, phosphate, ammonium, and pH—were determined using a multi-parameter digital water quality analyzer (YSI, ProDSS, Yellow Springs, USA) to ensure precision and consistency across all treatment groups.

### 2.4 Growth performance assessment

At the conclusion of the feeding trial, and following a 24-h fasting period to empty the gut contents, prawns were sampled from each treatment group for growth metrics analysis. Growth indices were calculated using standard equations as follows (Santos et al., 2019; Bagheri et al., 2023):

Weight gain (WG, g) = W _final (g)_ − W _initial (g)_

Weight gain rate (WGR, %) = 100 × (WG _(g)_ ÷ W _initial (g)_)

Specific growth rate (SGR, %/day) = 100 × [(ln. W _final (g)_) – ln. W _initial (g)_) ÷ experimental period _(days)_]

Feed conversion ratio (FCR) = feed intake _(g)_ ÷ WG _(g)_

Hepatosomatic index (HSI, %) = 100 × (W _hepatopancreas (g__)_÷ W _final (g)_)

Survival rate (SR, %) = 100 × (N _final_ ÷ N _initial_)

### 2.5 Hemolymph collection procedure

At the conclusion of the experimental period, prawns were subjected to a 24-h fasting protocol to ensure gut clearance, followed by a 30-min cold anesthesia treatment at 4 °C (Cole-Parmer, Laboratory Ice Chest, Vernon Hills, USA) to reduce handling stress. Subsequently, ten prawns from each replicate aquarium (also used for digestive enzyme analyses) were randomly selected for hemolymph extraction. Hemolymph was drawn using a 1 mL sterile syringe (BD, Monoject, Franklin Lakes, USA) preloaded with 0.4 mL of Alsever’s solution (Sigma-Aldrich, Catalogue No. A3551, St. Louis, USA) as an anticoagulant. The needle was inserted into the abdominal sinus at a 45° angle, and fluid was collected until it represented approximately 5 % of the individual’s body weight. Pooled hemolymph samples were transferred into sterile microtubes (Eppendorf, Safe-Lock Tubes, Hamburg, Germany). One portion was fixed in 10 % neutral buffered formalin (Sigma-Aldrich, Catalogue No. HT501128, St. Louis, USA) and refrigerated at 4 °C, while the remaining aliquot was immediately stored at −86 °C (Haier Biomedical, DW-86L728J, Qingdao, China) for subsequent biochemical analysis. Prior to analytical procedures, frozen samples were thawed and homogenized using a vortex mixer (IKA, MS 3 basic, Staufen, Germany) for 30–40 s (Yudiati et al., 2016; Bagheri et al., 2023; Bai et al., 2023).

### 2.6 Hematological analysis

Pooled hemolymph samples containing anticoagulant were subjected to centrifugation (Beckman Coulter, Allegra X-15R, Brea, USA) at 10,000 rpm and 4 °C for 30 min to separate the plasma fraction for biochemical evaluation. The supernatant was meticulously aspirated using a micropipette (Thermo Fisher Scientific, Finnpipette F1, Waltham, USA) and transferred into sterile 1.5 mL microcentrifuge tubes for subsequent analyses. A comprehensive panel of hematological biomarkers—including glucose, triglycerides, cholesterol, high-density lipoprotein (HDL), low-density lipoprotein (LDL), creatinine, urea, calcium, phosphorus, and uric acid—was quantified. Measurements were performed utilizing an automated biochemical analyzer (Siemens, Dimension EXL, Munich, Germany) alongside colorimetric assays, with all reagents procured as commercial diagnostic kits (Pars-Azmoon, Alborz, Iran) (Yudiati et al., 2016; Bagheri et al., 2023; Bai et al., 2023).

### 2.7 Immunological indices

Albumin (ALB) levels were quantified using a colorimetric assay based on bromocresol green (BCG) dye, with absorbance read at 630 nm. Total protein (TP) content was quantified using the Biuret reaction, measuring absorbance at 540 nm (Pars-Azmoon, Alborz, Iran). Cortisol (CORT) levels were assessed through enzyme-linked immunosorbent assay (ELISA) at 450 nm (Abcam, ab108665, Cambridge, UK). The activity of lysozyme (LYZ) was evaluated using *Micrococcus luteus* substrate (ZYS, Microbial ZYS-BANK ATCC No. 10,240, Tehran, Iran), recording absorbance at 530 nm, while phenoloxidase (PO) enzymatic activity was assessed by tracking DOPA-chrome formation at 490 nm (Abcam, ab111749, Cambridge, UK). Total hemocyte count (THC) was determined by mixing 100 μL of pooled hemolymph with an anticoagulant and an equal volume of 10 % neutral buffered formalin, followed by a 30-min incubation. Subsequently, hemocytes were enumerated using an Improved Neubauer hemocytometer (Hausser Scientific, Model 3100, Horsham, USA) under 40× magnification (Olympus, CX23, Tokyo, Japan). Differential hemocyte count (DHC) was performed by smearing 50 μL of hemolymph on glass slides (Menzel Gläser, Superfrost, Braunschweig, Germany), fixing in methanol (Merck, Catalogue No. 106,009, Darmstadt, Germany), staining with 10 % Giemsa (Sigma-Aldrich, Catalogue No. GS500, St. Louis, USA), and examining under 100× magnification. Activities of key enzymes including alkaline phosphatase (AKP), acid phosphatase (ACP), lactate dehydrogenase (LDH), alanine aminotransferase (ALT), and aspartate aminotransferase (AST) were measured employing an automated biochemical analyzer with commercial kits (Pars-Azmoon, Alborz, Iran), with absorbance read within the 340 – 410 nm wavelength range (Hindu et al., 2018; Wu et al., 2018; Bagheri et al., 2023).

### 2.8 Antioxidant parameters evaluation

For evaluating antioxidant parameters, ten prawns (previously utilized for HSI measurement) were randomly selected from each replicate aquarium. The hepatopancreas tissues were carefully excised and rinsed with double-distilled water chilled to 4 °C. The samples were then weighed and homogenized (Omni International, TH Tissue Homogenizer, Kennesaw, USA) in a Tris–HCl buffer (Sigma-Aldrich, Catalogue No. T5941, St. Louis, USA) at a ratio of 1:9 (w/v). Following homogenization, the pooled tissue suspensions were centrifuged at 10,000 rpm for 30 min at approximately 4 °C. The resulting supernatant was collected meticulously and stored at −86 °C until subsequent biochemical assays. Quantification of antioxidant biomarkers—including catalase (CAT), glutathione peroxidase (GPx), superoxide dismutase (SOD), total antioxidant capacity (T-AOC), and malondialdehyde (MDA)—was conducted using colorimetric assays with commercially available reagent kits (Jiancheng Bioengineering Institute, Catalogue Nos. A007–1, A001–1, A015–1, A003–1, A005–1, Nanjing, China). Measurements were performed on a microplate reader (BioTek, Synergy HTX, Winooski, USA), following the manufacturer’s standardized protocols (Cheng et al., 2005; Jiang et al., 2017; Bai et al., 2023).

### 2.9 Analysis of digestive enzyme activity

To examine variations in digestive enzyme function, prawns were subjected to a 24-h fasting period prior to sampling to ensure the elimination of residual feed. Ten individuals, (previously sampled for hemolymph) were randomly selected from each replicate aquarium. Their gastrointestinal tracts were dissected on ice under careful handling to minimize enzymatic degradation. The tissues were rinsed with chilled distilled water, weighed, and homogenized in a Tris–HCl buffer at a 1:9 wt-to-volume ratio. The homogenates were processed on ice for 30 s, followed by centrifugation at 10,000 rpm for 30 min at 4 °C. The supernatant was collected and stored at −86 °C until further enzymatic assays. Enzyme activities, including protease (366 nm), amylase (405 nm), lipase (550 nm), and cellulose (540 nm), were quantified through spectrophotometric analysis using commercial diagnostic kits (Pars-Azmoon, Alborz, Iran) (Cheng et al., 2005; Hindu et al., 2018; Adilah et al., 2022; Bai et al., 2023).

### 2.10 Analysis of intestinal microbiota

To evaluate the intestinal microbiota, ten prawns from each replicate aquarium (also utilized for gene expression analysis) were selected. The prawns were anesthetized using ice powder, and their abdomens were disinfected with aqueous ethanol (70 %). Intestinal tracts were aseptically excised, opened, and washed with sterile 0.9 % saline solution (Merck, Catalogue No. 1.06404.1000, Darmstadt, Germany) inside a laminar flow cabinet (Labconco, Purifier Logic+, Kansas City, USA) to maintain sterility. Intestinal contents were serially diluted in sterile saline from 10⁻¹ to 10⁻¹⁰. Subsequently, 100 μL aliquots from each dilution were plated onto Tryptic Soy Agar (TSA) (Sigma-Aldrich, Catalogue No. 22,091, St. Louis, USA) to determine total bacterial counts (TBC), and onto De Man–Rogosa–Sharpe (MRS) (Sigma-Aldrich, Catalogue No. 69,966, St. Louis, USA) agar for culturing lactic acid bacteria (LAB). Plates were incubated at 30–35 °C for 48 to 72 h, after which colony-forming units were enumerated using a colony counter (Starlab, Colony Counter, Hamburg, Germany). Results were presented as log colony-forming units per gram (log CFU/g) (Cheng et al., 2005; Hindu et al., 2018; Adilah et al., 2022; Bai et al., 2023).

### 2.11 Carcass composition evaluation

The carcass composition was assessed following the protocols outlined by AOAC (2016). Moisture content was measured by transferring a homogenized sample (previously sampled for hemolymph and digestive enzyme activity) into a pre-weighed and oven-dried Petri dish (SARSTEDT AG & Co. KG, Nümbrecht, Germany), then drying it in an oven set at 103 ± 2 °C until a constant weight was achieved. After drying, the sample was cooled in a desiccator (DWK Life Sciences, DURAN Desiccator, Wertheim, Germany) before reweighing. Crude protein content was quantified via the Kjeldahl nitrogen determination method (VELP Scientifica, UDK 149, Usmate Velate, Italy), while lipid extraction was performed using the Soxhlet apparatus (VELP Scientifica, SER 148/6 Soxhlet Extractor, Usmate Velate, Italy) with chloroform as solvent at 50–60 °C for a duration of 4 to 6 h. Ash content was assessed by subjecting the carcass to combustion in an electric muffle furnace (Nabertherm, LHT 02/17 LB, Lilienthal, Germany) at 550 °C for 12 h.

### 2.12 Profiling of amino acids and fatty acids

Amino acid profiling was performed following the protocol of Li et al. (2021). After freeze-drying, samples (previously utilized for intestinal microbiota and gene expression analysis) underwent hydrolysis in 6 M hydrochloric acid (Sigma-Aldrich, Catalogue No. H1758, St. Louis, USA), followed by sequential steps of dilution, evaporation, reconstitution, and filtration. Quantitative analysis was carried out using an amino acid analyzer (Shimadzu, UF-Amino Station, Kyoto, Japan). For fatty acid composition, a modified procedure adapted from Chen et al. (2020) was employed. A 100 mg sample was combined with 3 mL methanolic potassium hydroxide (Sigma-Aldrich, Catalogue No. 221,472, St. Louis, USA) and incubated at 72 °C for 20 min. To prepare the samples, 0.5 mL of methyl nonadecanoate (Sigma-Aldrich, Catalogue No. 74,208, St. Louis, USA) served as an internal standard. After cooling, 3 mL of hydrochloric acid in methanol (Sigma-Aldrich, Catalogue No. 160,758, St. Louis, USA) was added, followed by incubation at 72 °C for an additional 20 min. Subsequently, 1 mL of n-hexane (Sigma-Aldrich, Catalogue No. 296,090, St. Louis, USA) was incorporated, and the mixture was allowed to rest for 8 h. Thereafter, the supernatant was subjected to centrifugation at 10,000 rpm for 2 min, and fatty acid profiles were determined via gas chromatography (Agilent Technologies, 7890B GC System, Santa Clara, USA).

### 2.13 Evaluation of growth-, immunity-, and metabolism-associated gene expression

Total RNA was extracted by homogenizing 100 mg of the sample in 1 mL of RNA extraction reagent (Qiagen, RNeasy Mini Kit, Catalogue No. 74,104 Hilden, Germany), strictly adhering to the manufacturer's protocol. The integrity of extracted RNA was verified through agarose gel (Sigma-Aldrich, Catalogue No. A9539, St. Louis, USA) electrophoresis (Bio-Rad, PowerPac Basic Electrophoresis System, Hercules, USA), while purity was quantified by measuring the OD260/OD280 ratio using an ultra-micro spectrophotometer (NanoDrop Technologies, NanoDrop 2000c, Wilmington, USA). To eliminate genomic DNA contamination, samples were treated with DNase I (Thermo Fisher Scientific, Catalogue No. EN0521, Waltham, USA) and subsequently preserved at −86 °C until complementary DNA (cDNA) synthesis (Thermo Fisher Scientific, RevertAid First Strand cDNA Synthesis Kit, Catalogue No. K1622, Waltham, USA). Sequences of genes (related to growth: *LGR2, CHSs, RXR-S, EcR, CDA1*; related to immune function: *lectin, A2M, lysozyme, crustin, caspase*, and related to metabolism: *CPT-1, ELOV6, ACC, Δ9-D*) specific to *Macrobrachium nipponense* were obtained from the NCBI database, and corresponding primers were designed via the NCBI Primer-BLAST platform (refer to Table 2). Quantitative reverse transcription polymerase chain reaction (qRT-PCR) was conducted using SYBR Green qPCR Master Mix (Thermo Fisher Scientific, PowerUp SYBR Green Master Mix, Catalogue No. A25742, Waltham, USA) and a real-time PCR system (Bio-Rad, CFX96 Touch Real-Time PCR Detection System, Hercules, USA) to assess gene expression. The 25 μL reaction volume contained 12.5 μL of Master Mix, 2 μL of cDNA template, and 1 μL of each primer at 10 μM concentration. Thermal cycling conditions began with an initial denaturation at 95 °C for 30 s, followed by 40 amplification cycles comprising 95 °C denaturation for 30 s and annealing at 60 °C for 30 s. Relative gene expression was calculated via the 2^−ΔΔCt^ method, normalizing to β-actin expression, with each sample analyzed in triplicate (Santos et al., 2019; Yudiati et al., 2019; Bai et al., 2023).

**Table 2:** Primers utilized in quantitative real-time polymerase chain reaction (qPCR).

Gene	Primer sequences (5′−3′)	Product length (bp)	GenBank accession number
*LGR2*	F: CCTCCTCCTCCACGATAACC R: ACTCTTTCCAGACCTCGGTG	201	MT585155
*EcR*	F: CCATCGTTAAAAGAGCCGCA R: TTGAGATTGCCGAGGGTTCT	158	MH459143
*CHSs*	F: GGCCTAGTCCTCCTCATGAC R: GGCACGATGAAGCAGAAGAG	164	KP710198
*RXR-S*	F: AAGGGGACACGATGGGTTAG R: ATTGGACACACTGGGAGAGG	154	KC460324
*CDA1*	F: AGCGAACCCACTGAAGATGA R: TGTCCTTCCAGTCACAGGTC	196	MF360010
*A2M*	F: ACCGGACACCATCACAGAAT R: CTCTCTTCCAAGGACACGGT	219	MK439847
*Caspase*	F: TGGAGAACGAGCATTGCAAC R: CAAACTCCTGCAATGCCTGA	173	KU942381
*Crustin*	F: CTGTGACAGCAACCTCGATC R: ACGTGATGCTCCAGACAGAT	180	OM032597
*Lectin*	F: TGATGGAGGTCAACGGGTAC R: GTCCGGAGAGATAAAGGCCA	189	PP516428
*Lysozyme*	F: GGCAAGAACGTCTGTGGAAT R: CGAGGTCGCGATTCTTACAC	169	AY257550
*ELOV6*	F: TTCACGCTGAGCAAAGTTCC R: CGGAATTTCAGGGCCTTGAG	221	KU953779
*Δ^9^-D*	F: CACCCCTTCGCGTTGTTTTA R: ATGAGCCATCCCATATGCGA	163	KU922943
*CPT-1*	F: CGGACAAGATCGTGCACTTT R: AGCAGTAAGAGCACCAAGGT	191	KP690136
*ACC*	F: AGCACTCTAGGGTTGCTCTG R: TGGCATGGTAGAAGAAGGCA	207	KP690138
*β-actin*	F: AATCTTGCGGCATTCACGAG R: AGATAGAACCGCCGATCCAG	228	KY780298

*Note*: LGR2 = Leucine-rich repeat-containing G-protein-coupled receptor 2; EcR = Ecdysteroid receptor; CHSs = Chitin synthase; RXR-*S* = Retinoid X receptor; CDA1 = Cytidine deaminase 1; A2M = Alpha-2-macroglobulin; ELOV6 = Elongation of very long chain fatty acids protein 6; Δ9-*D* = Delta-9 desaturase; CPT-1 = Carnitine O-palmitoyltransferase; ACC = Acetyl-CoA carboxylase.

### 2.14 Statistical analysis

Statistical analyses were performed using SPSS software (IBM, version 27.0.1, Armonk, USA). The normality of data distribution was verified through the Kolmogorov-Smirnov test, while homogeneity of variances was assessed using Levene’s test. Differences among treatment means were determined via one-way analysis of variance (ANOVA), followed by Duncan’s multiple range test for post-hoc pairwise comparisons. Moreover, a follow-up trend analysis utilizing orthogonal polynomial regression was conducted to assess the presence of linear and/or quadratic patterns in the observed effect. Statistical significance was accepted at *P* < 0.05. Results are expressed as mean ± standard deviation.

## 3 Results

### 3.1 Water quality parameters

The inclusion of sodium alginate had no significant effect on most water quality parameters, including temperature, pH, ammonium, nitrite, nitrate, phosphate, and total dissolved solids (*P* > 0.05, Table 3). However, dissolved oxygen levels increased significantly with sodium alginate supplementation (*P* < 0.05), rising from 7.12 mg/L in the control to 7.80 mg/L at 4.0 g/kg. The increase was significant in the 2.0 and 4.0 g/kg groups compared to the control, while the 0.5 and 1.0 g/kg groups showed intermediate values that did not differ significantly from either the control or the highest level.

**Table 3:** Influence of dietary sodium alginate on *Macrobrachium nipponense*: an assessment of water quality indicators over a 60-day feeding period. Values represent mean ± standard deviation (*M* ± SD, *n* = 3). Statistically significant differences among groups are indicated by distinct superscript letters within each row (*P* < 0.05).

Parameters			Sodium alginate (g/kg)			ANOVA	Linear	Quadratic
	0.0	0.5	1.0	2.0	4.0	*P*-value (F)	*P*-value (R^2^)	*P*-value (R^2^)
Temperature (°C)	25.87±0.55	26.09±0.59	24.84±0.35	24.77±0.40	24.68±0.61	0.882 (0.336)	0.137 (0.575)	0.234 (0.765)
pH	7.38±0.23	7.44±0.33	6.64±0.39	7.15±0.21	7.45±0.18	0.603 (0.711)	0.783 (0.029)	0.579 (0.421)
Dissolved oxygen (mg/L)	7.12±0.14^d^	7.55±0.04^ab^	7.27±0.09^c^	7.44±0.06^b^	7.80±0.05^a^	<0.001 (32.607)	0.032 (0.975)	0.024 (0.980)
Ammonium (mg/L)	0.48±0.05	0.44±0.04	0.47±0.05	0.44±0.06	0.43±0.03	0.523 (0.947)	0.379 (0.261)	0.738 (0.262)
Nitrite (mg/L)	0.13±0.03	0.13±0.03	0.12±0.02	0.12±0.01	0.13±0.03	0.565 (0.884)	0.169 (0.521)	0.198 (0.802)
Nitrate (mg/L)	0.15±0.01	0.15±0.02	0.16±0.02	0.16±0.02	0.15±0.01	0.422 (1.273)	0.612 (0.115)	0.189 (0.811)
Phosphate (mg/L)	0.03±0.02	0.03±0.01	0.02±0.02	0.03±0.01	0.02±0.01	0.491 (0.930)	0.302 (0.742)	0.181 (0.826)
Total dissolved solids (mg/L)	168.09±3.99	171.82±4.06	174.90±3.49	163.61±3.58	161.87±3.50	0.334 (1.599)	0.169 (0.520)	0.454 (0.546)

### 3.2 Growth performance

**Fig. 1:**
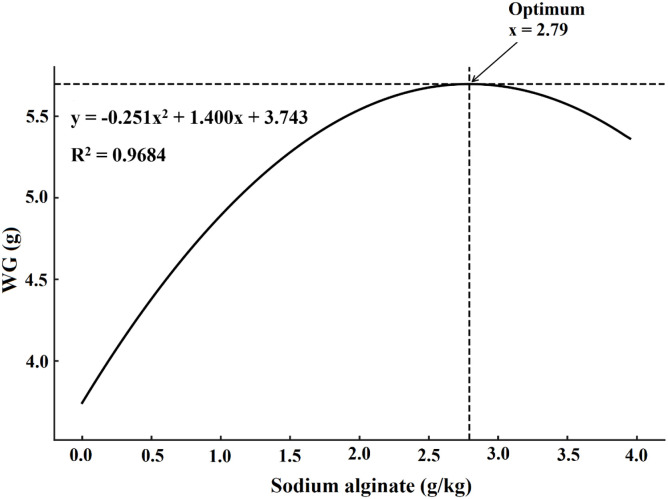
Quadratic regression analysis of weight gain (WG) in *Macrobrachium nipponense* subjected to different dietary sodium alginate levels after a 60-day feeding trial.

**Fig. 2:**
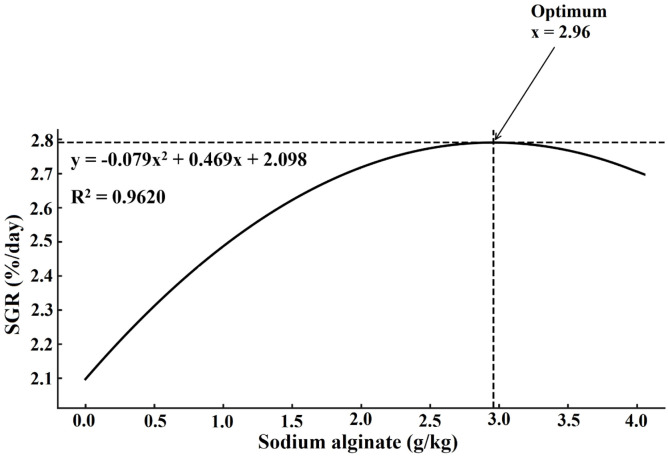
Quadratic regression analysis of specific growth rate (SGR) in *Macrobrachium nipponense* subjected to different dietary sodium alginate levels after a 60-day feeding trial.

**Fig. 3:**
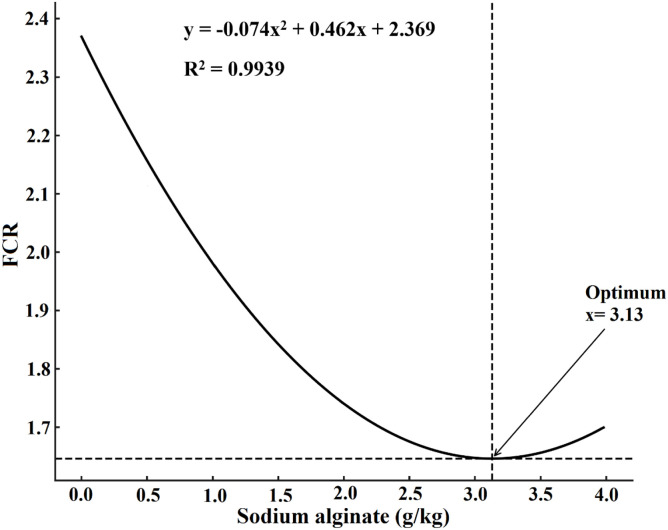
Quadratic regression analysis of feed conversion ratio (FCR) in *Macrobrachium nipponense* subjected to different dietary sodium alginate levels after a 60-day feeding trial.

Growth performance metrics improved significantly with increasing sodium alginate levels (*P* < 0.05, Table 4). FW, WG, WGR, and SGR were all significantly higher in the 2.0 and 4.0 g/kg groups compared to the control and lower-dose groups, while FCR decreased significantly in these higher supplementation groups. SR reached 100 % in the 2.0 and 4.0 g/kg groups, which was significantly higher than the control (94.77 %). Regression analyses indicated significant quadratic trends for WG, SGR, and FCR (Figs. 1, 2 and 3).

**Table 4:** Influence of dietary sodium alginate on *Macrobrachium nipponense*: an assessment of growth performance over a 60-day feeding period. Values represent mean ± standard deviation (*M* ± SD, *n* = 3). Statistically significant differences among groups are indicated by distinct superscript letters within each row (*P* < 0.05).

Parameters			Sodium alginate (g/kg)			ANOVA	Linear	Quadratic
	0.0	0.5	1.0	2.0	4.0	*P*-value (F)	*P*-value (R^2^)	*P*-value (R^2^)
FW (g)	5.05±0.36^e^	5.94±0.35^d^	6.23±0.3^c^	6.81±0.39^b^	6.90±0.46^a^	<0.001 (256.08)	0.043 (0.859)	0.019 (0.981)
WG (g)	3.62±0.33^e^	4.49±0.25^d^	4.76±0.42^c^	5.33±0.53^b^	5.42±0.47^a^	<0.001 (244.30)	0.011 (0.988)	0.032 (0.968)
WGR (%)	246.25±27.41^d^	305.44±23.91^c^	323.80±24.40^b^	362.58±29.88^a^	368.71±31.64^a^	<0.001 (131.41)	0.018 (0.989)	0.019 (0.980)
SGR (%/day)	2.06±0.16^d^	2.36±0.11^c^	2.45±0.17^b^	2.57±0.15^a^	2.64±0.22^a^	<0.001 (178.26)	0.041 (0.855)	0.038 (0.962)
FCR	2.39±0.07^a^	2.12±0.08^b^	1.99±0.05^c^	1.75±0.09^d^	1.69±0.08^e^	<0.001 (396.33)	0.045 (0.779)	0.006 (0.994)
HSI (%)	4.67±0.58^c^	4.98±0.40^b^	5.09±0.51^ab^	5.10±0.44^ab^	5.11±0.39^a^	<0.001 (230.627)	0.038 (0.863)	0.021 (0.907)
SR (%)	94.77±3.52^d^	95.83±2.60^c^	98.55±1.30^b^	100.00±0.00^a^	100.00±0.00^a^	<0.001 (420.78)	0.033 (0.962)	0.026 (0.974)

*Note*: FW = Final weight; WG = Weight gain; WGR = Weight gain rate; SGR = Specific growth rate; FCR = Feed conversion ratio; HSI = Hepatosomatic index; SR = Survival rate.

### 3.3 Hemato-biochemical indices

As shown in Table 5, sodium alginate supplementation significantly reduced levels of urea, uric acid, glucose, and creatinine compared to the control (*P* < 0.05). The reductions were most pronounced in the 2.0 and 4.0 g/kg groups. Conversely, HDL and LDL levels increased significantly with sodium alginate inclusion (*P* < 0.05), particularly in the higher supplementation groups. Calcium and phosphorus levels did not differ significantly among treatments (*P* > 0.05).

**Table 5:** Influence of dietary sodium alginate on *Macrobrachium nipponense*: an assessment of hemato-biochemical parameters over a 60-day feeding period. Values represent mean ± standard deviation (*M* ± SD, *n* = 3). Statistically significant differences among groups are indicated by distinct superscript letters within each row (*P* < 0.05).

Parameters			Sodium alginate (g/kg)			ANOVA	Linear	Quadratic
	0.0	0.5	1.0	2.0	4.0	*P*-value (F)	*P*-value (R^2^)	*P*-value (R^2^)
Urea (mg/dL)	18.94±0.53^a^	17.31±0.47^b^	16.97±0.32^c^	16.78±0.47^d^	16.22±0.41^e^	<0.001 (36.220)	0.036 (0.861)	0.030 (0.903)
Uric acid (mg/dL)	1.91±0.29^a^	1.78±0.16^b^	1.77±0.20^b^	1.70±0.18^bc^	1.63±0.15^c^	<0.001 (17.336)	0.028 (0.842)	0.028 (0.842)
Glucose (mg/dL)	55.13±0.63^ab^	56.01±0.37^a^	54.91±0.46^b^	53.94±0.32^c^	50.53±0.37^d^	<0.001 (110.015)	0.032 (0.961)	0.020 (0.921)
Creatinine (mg/dL)	0.35±0.03^a^	0.33±0.01^b^	0.31±0.03^c^	0.30±0.02^c^	0.25±0.01^d^	0.007 (8.146)	0.002 (0.972)	0.002 (0.976)
Calcium (mg/dL)	82.85±2.05	84.39±1.99	78.79±1.98	79.32±1.50	80.03±1.42	0.786 (0.602)	0.138 (0.574)	0.324 (0.676)
Phosphorus (mg/dL)	18.75±0.77	19.06±0.63	18.56±0.84	19.06±0.61	18.76±0.69	0.937 (0.094)	0.243 (0.412)	0.352 (0.648)
Cholesterol (mg/dL)	49.61±0.53^a^	49.94±0.44^a^	45.88±0.49^b^	45.61±0.41^b^	44.78±0.39^c^	<0.001 (123.002)	0.002 (0.979)	0.001 (0.980)
Triglycerides (mg/dL)	80.24±0.75^a^	76.70±0.58^b^	73.9 ± 0.41^c^	75.39±0.45^bc^	69.32±0.41^d^	<0.001 (138.524)	0.032 (0.828)	0.029 (0.903)
HDL (mg/dL)	15.46±0.39^d^	16.80±0.36^c^	17.39±0.35^b^	17.53±0.38^b^	19.03±0.40^a^	<0.001 (24.145)	0.026 (0.847)	0.023 (0.866)
LDL (mg/dL)	8.17±0.16^d^	8.63±0.22^c^	9.15±0.20^b^	9.77±0.31^a^	9.83±0.25^a^	<0.001 (51.119)	0.014 (0.911)	0.009 (0.990)

*Note*: HDL = High-density lipoprotein; LDL = Low-density lipoprotein.

### 3.4 Hemato-immune responses

Albumin and total protein concentrations increased significantly with dietary sodium alginate (*P* < 0.05, Table 6), with the highest values in the 4.0 g/kg group. Cortisol levels decreased significantly across supplementation groups compared to the control (*P* < 0.05). Lysozyme and phenoloxidase activities were significantly higher in the 2.0 and 4.0 g/kg groups than in the control (*P* < 0.05). Total and differential hemocyte counts (THC, GC, SGC, HC) were also significantly elevated in the 2.0 and 4.0 g/kg treatments compared to the control (*P* < 0.05, Table 7).

**Table 6:** Influence of dietary sodium alginate on *Macrobrachium nipponense*: an assessment of hemato-immune indices over a 60-day feeding period. Values represent mean ± standard deviation (*M* ± SD, *n* = 3). Statistically significant differences among groups are indicated by distinct superscript letters within each row (*P* < 0.05).

Parameters			Sodium alginate (g/kg)			ANOVA	Linear	Quadratic
	0.0	0.5	1.0	2.0	4.0	*P*-value (F)	*P*-value (R^2^)	*P*-value (R^2^)
ALB (g/dL)	1.78±0.07^c^	1.91±0.04^b^	1.97±0.04^ab^	1.94±0.06^ab^	2.13±0.09^a^	<0.001 (67.133)	0.043 (0.792)	0.039 (0.807)
TP (g/dL)	7.26±0.15^d^	8.26±0.10^c^	8.33±0.29^c^	9.40±0.19^b^	9.93±0.22^a^	<0.001 (212.329)	0.036 (0.841)	0.025 (0.930)
CORT (ng/mL)	19.79±0.75^a^	17.3 ± 0.53^b^	16.17±0.65^c^	15.73±0.62^d^	14.54±0.37^e^	<0.001 (105.014)	0.045 (0.746)	0.030 (0.917)
LYZ (U/min/mL)	16.69±0.32^d^	17.99±0.37^c^	20.01±0.38^b^	20.18±0.54^b^	22.73±0.52^a^	<0.001 (274.619)	0.013 (0.905)	0.008 (0.942)
PO (U/min/mg protein)	0.78±0.03^d^	0.86±0.06^c^	0.94±0.04^b^	0.92±0.04^b^	1.14±0.05^a^	<0.001 (20.102)	0.010 (0.930)	0.008 (0.944)

*Note*: ALB = Albumin; TP = Total protein; CORT = Cortisol; LYZ = Lysozyme; PO = Phenoloxidase.

**Table 7:** Influence of dietary sodium alginate on *Macrobrachium nipponense*: an assessment of cell-mediated immune responses over a 60-day feeding period. Values represent mean ± standard deviation (*M* ± SD, *n* = 3). Statistically significant differences among groups are indicated by distinct superscript letters within each row (*P* < 0.05).

Parameters			Sodium alginate (g/kg)			ANOVA	Linear	Quadratic
	0.0	0.5	1.0	2.0	4.0	*P*-value (F)	*P*-value (R^2^)	*P*-value (R^2^)
THC (×10^5^ cells/mL)	107.78±2.19^e^	121.54±1.87^d^	138.6 ± 1.89^c^	148.96±1.22^b^	155.28±2.37^a^	<0.001 (280.172)	0.037 (0.855)	0.014 (0.986)
GC (×10^5^ cells/mL)	13.52±1.59^e^	20.48±2.12^d^	22.67±1.68^c^	25.32±2.14^b^	28.47±2.10^a^	<0.001 (51.249)	0.042 (0.774)	0.025 (0.910)
SGC (×10^5^ cells/mL)	41.44±3.18^e^	47.53±2.49^d^	57.08±1.47^c^	62.89±1.84^b^	64.04±1.85^a^	<0.001 (88.622)	0.035 (0.867)	0.016 (0.984)
HC (×10^5^ cells/mL)	49.75±3.19^e^	56.19±3.07^d^	58.21±2.43^c^	64.48±2.58^b^	66.33±2.37^a^	<0.001 (105.943)	0.038 (0.829)	0.014 (0.986)

*Note*: THC = Total hemocyte count; GC = Granular cells; SGC = Semi-granular cells; HC = Hyaline cells.

### 3.5 Enzymatic activity in hemolymph

ALT and AST decreased significantly with sodium alginate inclusion, from 28.78 to 19.22 U/L and 79.56 to 67.68 U/L, respectively (*P* < 0.05, Table 8). LDH also decreased from 709.69 to 687.22 U/L. No significant changes were observed in AKP and ACP activities (*P* > 0.05).

**Table 8:** Influence of dietary sodium alginate on *Macrobrachium nipponense*: an assessment of enzymatic hemolymph parameters over a 60-day feeding period. Values represent mean ± standard deviation (*M* ± SD, *n* = 3). Statistically significant differences among groups are indicated by distinct superscript letters within each row (*P* < 0.05).

Parameters			Sodium alginate (g/kg)			ANOVA	Linear	Quadratic
	0.0	0.5	1.0	2.0	4.0	*P*-value (F)	*P*-value (R^2^)	*P*-value (R^2^)
ALT (U/L)	28.78±1.41^a^	24.58±0.94^b^	25.35±0.85^c^	21.49±0.6^d^	19.22±0.67^e^	<0.001 (36.017)	0.022 (0.865)	0.011 (0.930)
AST (U/L)	79.56±1.30^a^	77.56±1.11^b^	72.34±1.21^c^	71.29±0.95^c^	67.68±1.27^d^	<0.001 (40.159)	0.024 (0.855)	0.032 (0.947)
AKP (U/L)	184.36±1.82	186.03±1.58	184.16±2.16	187.15±1.92	188.81±2.11	0.127 (4.620)	0.087 (0.677)	0.186 (0.815)
ACP (U/L)	294.32±2.42	301.05±2.58	297.73±2.07	299.92±1.92	302.58±1.13	0.135 (3.918)	0.079 (0.694)	0.781 (0.219)
LDH (U/L)	709.69±5.41^b^	717.41±6.1^a^	702.16±4.81^c^	698.04±3.7^d^	687.22±3.74^e^	0.005 (8.224)	0.010 (0.936)	0.018 (0.872)

*Note*: ALT = Alanine aminotransferase; AST = Aspartate aminotransferase; AKP = Alkaline phosphatase; ACP = Acid phosphatase; LDH = Lactate dehydrogenase.

### 3.6 Antioxidant status

As expressed in Table 9, dietary sodium alginate significantly increased T-AOC and CAT activities in the higher supplementation groups (2.0 and 4.0 g/kg) compared to the control (*P* < 0.05). MDA levels decreased significantly with increasing alginate inclusion (*P* < 0.05). SOD activity declined significantly in sodium alginate groups, while GPx activity did not differ significantly among treatments (*P* > 0.05).

**Table 9:** Influence of dietary sodium alginate on *Macrobrachium nipponense*: an assessment of hepatopancreatic antioxidant activities over a 60-day feeding period. Values represent mean ± standard deviation (*M* ± SD, *n* = 3). Statistically significant differences among groups are indicated by distinct superscript letters within each row (*P* < 0.05).

Parameters			Sodium alginate (g/kg)			ANOVA	Linear	Quadratic
	0.0	0.5	1.0	2.0	4.0	*P*-value (F)	*P*-value (R^2^)	*P*-value (R^2^)
T-AOC (U/mg protein)	6.02±0.22^d^	5.73±0.28^e^	6.65±0.22^c^	7.18±0.43^b^	7.46±0.35^a^	<0.001 (36.412)	0.041 (0.859)	0.044 (0.852)
SOD (U/mg protein)	9.59±0.37^a^	8.86±0.47^b^	8.56±0.47^c^	8.34±0.26^c^	7.30±0.39^d^	<0.001 (19.025)	0.011 (0.914)	0.010 (0.917)
GPx (U/mg protein)	35.02±2.36	33.98±1.81	32.08±1.91	32.5 ± 2.12	34.98±2.32	0.172 (3.605)	0.797 (0.225)	0.141 (0.857)
CAT (U/mg protein)	19.33±0.64^a^	14.57±1.05^b^	14.51±1.01^b^	13.92±0.62^c^	13.75±0.46^c^	<0.001 (48.115)	0.033 (0.879)	0.031 (0.883)
MDA (nmol/mg protein)	11.36±0.51^a^	10.08±0.38^b^	9.13±0.53^c^	9.08±0.76^d^	8.26±0.81^e^	<0.001 (22.218)	0.009 (0.947)	0.035 (0.897)

*Note*: T-AOC = Total antioxidant capacity; SOD = Superoxide dismutase; GPx = Glutathione peroxidase; CAT = Catalase; MDA = Malondialdehyde.

### 3.7 Digestive enzyme activity

Protease, lipase, cellulase, and amylase activities increased significantly with sodium alginate supplementation (*P* < 0.05, Table 10). The 2.0 and 4.0 g/kg groups exhibited significantly higher enzyme activities compared to the control and lower supplementation levels (*P* < 0.05). Amylase rose from 2.33 to 3.61 U/mg protein, lipase from 1.02 to 1.32 U/mg, protease from 1.72 to 2.08 U/mg, and cellulase from 0.19 to 0.38 U/mg protein.

**Table 10:** Influence of dietary sodium alginate on *Macrobrachium nipponense*: an assessment of digestive enzyme activities over a 60-day feeding period. Values represent mean ± standard deviation (*M* ± SD, *n* = 3). Statistically significant differences among groups are indicated by distinct superscript letters within each row (*P* < 0.05).

Parameters			Sodium alginate (g/kg)			ANOVA	Linear	Quadratic
	0.0	0.5	1.0	2.0	4.0	*P*-value (F)	*P*-value (R^2^)	*P*-value (R^2^)
Protease (U/mg protein)	1.72±0.04^c^	1.89±0.09^b^	1.92±0.05^b^	2.04±0.05^a^	2.08±0.07^a^	<0.001 (39.003)	0.020 (0.962)	0.039 (0.887)
Lipase (U/mg protein)	1.02±0.02^e^	1.13±0.03^d^	1.19±0.07^c^	1.24±0.03^b^	1.32±0.06^a^	<0.001 (32.117)	0.009 (0.929)	0.005 (0.945)
Cellulase (U/mg protein)	0.19±0.02^d^	0.27±0.04^c^	0.28±0.03^c^	0.31±0.05^b^	0.38±0.04^a^	<0.001 (36.218)	0.016 (0.886)	0.007 (0.931)
Amylase (U/mg protein)	2.33±0.03^e^	2.75±0.06^d^	2.81±0.04^c^	3.14±0.04^b^	3.61±0.09^a^	<0.001 (43.129)	0.005 (0.949)	0.002 (0.976)

### 3.8 Intestinal microflora

TBC decreased from 8.20 to 7.28 log CFU/g, while LAB counts increased from 1.53 to 2.11 log CFU/g in groups receiving 2 and 4.0 g/kg of dietary sodium alginate supplementation (*P* < 0.05, Table 11).

**Table 11:** Influence of dietary sodium alginate on *Macrobrachium nipponense*: an assessment of intestinal microflora over a 60-day feeding period. Values represent mean ± standard deviation (*M* ± SD, *n* = 3). Statistically significant differences among groups are indicated by distinct superscript letters within each row (*P* < 0.05).

Parameters			Sodium alginate (g/kg)			ANOVA	Linear	Quadratic
	0.0	0.5	1.0	2.0	4.0	*P*-value (F)	*P*-value (R^2^)	*P*-value (R^2^)
TBC (log_10_ CFU/g)	8.20±0.31^a^	7.95±0.12^b^	8.04±0.16^c^	7.49±0.18^d^	7.28±0.24^e^	<0.001 (18.198)	0.032 (0.828)	0.027 (0.896)
LAB (log_10_ CFU/g)	1.53±0.03^e^	1.62±0.07^d^	1.71±0.10^c^	1.82±0.05^b^	2.11±0.07^a^	0.027 (4.636)	0.007 (0.937)	0.001 (0.999)

*Note*: TBC = Total bacteria count; LAB = Lactic acid bacteria.

### 3.9 Whole-body composition

Moisture content declined from 69.45 % to 66.53 %, while crude protein increased from 18.97 % to 21.82 %, and crude lipid from 6.77 % to 8.83 % in groups receiving 4.0 g/kg of dietary sodium alginate supplementation compared to the control (*P* < 0.05, Table 12). Ash content decreased from 4.79 % to 3.43 % at 4.0 g/kg of sodium alginate supplementation.

**Table 12:** Influence of dietary sodium alginate on *Macrobrachium nipponense*: an assessment of whole-body proximate composition over a 60-day feeding period. Values represent mean ± standard deviation (*M* ± SD, *n* = 3). Statistically significant differences among groups are indicated by distinct superscript letters within each row (*P* < 0.05).

Parameters			Sodium alginate (g/kg)			ANOVA	Linear	Quadratic
	0.0	0.5	1.0	2.0	4.0	*P*-value (F)	*P*-value (R^2^)	*P*-value (R^2^)
Moisture (%)	69.45±0.49^a^	68.30±0.24^b^	67.33±0.38^c^	68.10±0.54^b^	66.53±0.57^d^	<0.001 (70.127)	0.028 (0.930)	0.031 (0.897)
Crude protein (%)	18.97±0.25^d^	19.01±0.38^d^	19.31±0.38^c^	19.74±0.40^b^	21.82±0.43^a^	<0.001 (55.003)	0.011 (0.912)	0.004 (0.962)
Crude lipid (%)	6.77±0.27^d^	8.03±0.17^c^	8.42±0.14^b^	8.75±0.23^a^	8.83±0.18^a^	<0.001 (48.189)	0.002 (0.983)	0.005 (0.944)
Ash (%)	4.79±0.15^a^	4.66±0.21^ab^	4.54±0.28^b^	4.11±0.23^c^	3.43±0.27^d^	<0.001 (33.627)	0.026 (0.850)	0.030 (0.881)

### 3.10 Amino acid profile

The total EAA content increased in sodium alginate-fed groups from 34.58 % to 39.23 %, while ΣNEAA declined from 43.95 % to 40.57 % (*P* < 0.05, Table 13). Increases were also seen in arginine (4.96 to 5.58 %), histidine (3.10 to 3.64 %), and lysine (4.62 to 5.26 %) at the 2.0 and 4.0 g/kg groups than in the control (*P* < 0.05).

**Table 13:** Influence of dietary sodium alginate on *Macrobrachium nipponense*: an assessment of body amino acid profiles (%, dry matter) over a 60-day feeding period. Values represent mean ± standard deviation (*M* ± SD, *n* = 3). Statistically significant differences among groups are indicated by distinct superscript letters within each row (*P* < 0.05).

Parameters			Sodium alginate (g/kg)			ANOVA	Linear	Quadratic
	0.0	0.5	1.0	2.0	4.0	*P*-value (F)	*P*-value (R^2^)	*P*-value (R^2^)
EAA								
Arginine	4.96±0.04^c^	5.11±0.06^bc^	5.16±0.09^bc^	5.43±0.06^b^	5.58±0.05^a^	<0.001 (300.937)	0.008 (0.930)	0.004 (0.947)
Histidine	3.10±0.08^e^	3.16±0.04^d^	3.34±0.02^c^	3.45±0.04^b^	3.64±0.06^a^	<0.001 (221.882)	0.010 (0.990)	0.031 (0.969)
Isoleucine	2.25±0.04^c^	2.24±0.07^c^	2.38±0.05^b^	2.40±0.09^b^	2.48±0.07^a^	<0.001 (22.26)	0.019 (0.987)	0.20 (0.978)
Leucine	4.07±0.09^d^	4.22±0.06^c^	4.28±0.08^c^	4.49±0.06^b^	4.64±0.08^a^	<0.001 (204.163)	0.040 (0.857)	0.037 (0.961)
Lysine	4.62±0.13^c^	4.99±0.08^b^	5.04±0.07^b^	5.22±0.04^a^	5.26±0.08^a^	<0.001 (57.835)	0.044 (0.780)	0.005 (0.996)
Methionine	3.14±0.04^c^	3.29±0.06^b^	3.26±0.07^b^	3.31±0.06^ab^	3.44±0.07^a^	<0.001 (293.754)	0.037 (0.865)	0.021 (0.909)
Phenylalanine	5.13±0.11^d^	5.25±0.07^c^	5.26±0.06^c^	5.43±0.09^b^	5.55±0.07^a^	<0.001 (109.491)	0.026 (0.843)	0.025 (0.851)
Threonine	1.92±0.07^c^	2.17±0.08^b^	2.21±0.05^ab^	2.25±0.07^ab^	2.30±0.10^a^	<0.001 (23.962)	0.031 (0.963)	0.019 (0.924)
Tryptophan	1.97±0.06^ab^	1.93±0.04^b^	1.94±0.07^b^	1.99±0.03^ab^	2.04±0.05^a^	<0.001 (384.723)	0.013 (0.972)	0.012 (0.976)
Valine	3.42±0.09^e^	3.81±0.12^c^	3.77±0.05^d^	4.25±0.06^b^	4.30±0.07^a^	<0.001 (472.134)	0.032 (0.975)	0.024 (0.980)
ΣEAA	34.58±0.44	36.17±0.48	36.64±0.85	38.22±0.89	39.23±0.75	<0.001 (199.970)	0.008 (0.979)	0.007 (0.980)
NEAA								
Alanine	7.28±0.09^a^	7.09±0.08^c^	7.17±0.06^b^	6.94±0.06^d^	6.86±0.08^e^	<0.001 (23.966)	0.031 (0.829)	0.027 (0.905)
Aspartic acid	6.85±0.07^a^	6.63±0.13^b^	6.57±0.11^bc^	6.52±0.18^c^	6.40±0.12^d^	<0.001 (384.727)	0.026 (0.847)	0.022 (0.868)
Cysteine	3.16±0.05^a^	3.14±0.09^a^	3.10±0.07^ab^	3.09±0.08^ab^	2.99±0.04^b^	<0.001 (347.511)	0.035 (0.843)	0.024 (0.938)
Glutamic acid	10.53±0.16^a^	10.38±0.11^b^	10.19±0.18^c^	10.02±0.16^d^	9.89±0.16^e^	<0.001 (199.984)	0.042 (0.755)	0.023 (0.912)
Glycine	5.94±0.15^a^	5.50±0.07^b^	5.37±0.09^c^	5.35±0.12^c^	5.22±0.09^d^	<0.001 (107.750)	0.012 (0.907)	0.007 (0.943)
Proline	4.54±0.09^a^	4.39±0.05^b^	4.31±0.04^b^	4.24±0.09^c^	4.05±0.09^d^	0.002 (11.552)	0.040 (0.776)	0.024 (0.915)
Serine	2.18±0.06^a^	2.17±0.08^a^	2.09±0.04^b^	2.04±0.03^b^	2.06±0.05^b^	<0.001 (368.135)	0.038 (0.860)	0.019 (0.981)
Tyrosine	3.47±0.09^a^	3.39±0.09^b^	3.28±0.04^c^	3.07±0.06^d^	3.07±0.03^d^	<0.001 (301.562)	0.010 (0.926)	0.007 (0.949)
ΣNEAA	43.95±0.58^a^	42.69±0.98^b^	42.08±0.97^c^	41.27±1.07^d^	40.57±1.15^e^	<0.001 (17.085)	0.018 (0.889)	0.012 (0.935)
ΣEAA/ΣNEAA	0.79±0.02^d^	0.85±0.03^c^	0.87±0.04^c^	0.93±0.03^b^	0.97±0.03^a^	<0.001 (147.168)	0.034 (0.962)	0.027 (0.978)

*Note*: EAA = Essential amino acids; NEAA = Non-essential amino acids.

### 3.11 Fatty acid profile

ΣSFA decreased from 22.92 % to 16.16 % in sodium alginate treatments, while ΣMUFA and ΣPUFA increased from 45.32 % to 49.23 % and 31.76 % to 34.61 %, respectively (*P* < 0.05, Table 14). Notably, DHA rose from 3.52 % to 4.11 %, and EPA from 4.40 % to 4.85 % in the 2.0 and 4.0 g/kg groups compared to the control (*P* < 0.05).

**Table 14:** Influence of dietary sodium alginate on *Macrobrachium nipponense*: an assessment of body fatty acid compositions (%, dry matter) over a 60-day feeding period. Values represent mean ± standard deviation (*M* ± SD, *n* = 3). Statistically significant differences among groups are indicated by distinct superscript letters within each row (*P* < 0.05).

Parameters			Sodium alginate (g/kg)			ANOVA	Linear	Quadratic
	0.0	0.5	1.0	2.0	4.0	*P*-value (F)	*P*-value (R^2^)	*P*-value (R^2^)
C14:0	4.74±0.05^a^	4.70±0.06^ab^	4.66±0.05^b^	4.59±0.03^c^	4.51±0.02^d^	0.001 (13.040)	0.011 (0.923)	0.008 (0.950)
C15:0	0.67±0.07^a^	0.65±0.04^ab^	0.63±0.02^ab^	0.62±0.04^ab^	0.60±0.03^b^	0.001 (12.429)	0.019 (0.887)	0.014 (0.934)
C16:0	11.99±0.10^a^	11.50±0.06^b^	8.95±0.07^c^	7.74±0.07^d^	6.62±0.07^e^	<0.001 (108.265)	0.039 (0.862)	0.018 (0.983)
C17:0	0.49±0.06^a^	0.48±0.04^a^	0.47±0.03^ab^	0.44±0.03^b^	0.41±0.06^c^	0.003 (10.158)	0.033 (0.961)	0.028 (0.976)
C18:0	3.98±0.06^a^	3.30±0.03^b^	3.26±0.03^bc^	3.27±0.04^bc^	3.15±0.07^c^	0.001 (12.388)	0.041 (0.779)	0.023 (0.914)
C20:0	0.49±0.04^a^	0.47±0.03^ab^	0.45±0.03^b^	0.46±0.04^ab^	0.44±0.05^b^	0.002 (11.794)	0.013 (0.906)	0.008 (0.944)
C22:0	0.29±0.04^a^	0.26±0.02^bc^	0.27±0.03^b^	0.25±0.02^bc^	0.23±0.02^c^	0.004 (10.342)	0.041 (0.758)	0.022 (0.910)
C23:0	0.27±0.02^a^	0.25±0.03^ab^	0.24±0.04^ab^	0.21±0.03^b^	0.20±0.06^b^	<0.001 (14.481)	0.034 (0.847)	0.025 (0.940)
ΣSFA	22.92±0.22^a^	21.61±0.17^b^	18.93±0.14^c^	17.58±0.19^d^	16.16±0.15^e^	<0.001 (426.222)	0.027 (0.850)	0.021 (0.867)
C16:n	8.14±0.10^d^	8.57±0.07^c^	8.76±0.05^b^	8.83±0.05^b^	9.18±0.08^a^	<0.001 (242.603)	0.032 (0.832)	0.029 (0.903)
C17:n	1.67±0.04^c^	1.68±0.05^bc^	1.72±0.03^b^	1.75±0.04^ab^	1.77±0.05^a^	<0.001 (559.733)	0.007 (0.977)	0.006 (0.979)
C18:n9	30.66±0.09^e^	31.08±0.17^d^	32.68±0.08^c^	32.80±0.11^b^	32.89±0.07^a^	<0.001 (396.263)	0.015 (0.974)	0.011 (0.978)
C20:n9	3.34±0.04^c^	3.39±0.05^c^	3.55±0.04^b^	3.63±0.05^b^	3.72±0.05^a^	<0.001 (152.610)	0.034 (0.973)	0.025 (0.981)
C22:n9	1.51±0.03^c^	1.59±0.03^b^	1.58±0.03^b^	1.61±0.03^b^	1.67±0.03^a^	<0.001 (505.475)	0.032 (0.965)	0.018 (0.929)
ΣMUFA	45.32±0.18^e^	46.31±0.13^d^	48.29±0.19^c^	48.62±0.15^b^	49.23±0.13^a^	<0.001 (0.000)	0.009 (0.925)	0.005 (0.951)
C18:2n6	17.06±0.06^c^	16.91±0.05^d^	17.18±0.07^c^	17.46±0.11^b^	17.68±0.07^a^	<0.001 (333.462)	0.011 (0.992)	0.030 (0.996)
C18:3n6	0.84±0.02^d^	0.87±0.03^c^	0.89±0.03^c^	1.02±0.03^b^	1.11±0.04^a^	<0.001 (0.000)	0.020 (0.985)	0.019 (0.980)
C18:3n3	1.46±0.03^d^	1.52±0.02^c^	1.53±0.02^c^	1.59±0.03^b^	1.67±0.03^a^	<0.001 (50.477)	0.038 (0.860)	0.036 (0.863)
C20:2n	3.59±0.05^d^	3.67±0.03^c^	3.86±0.04^b^	3.99±0.05^a^	3.97±0.06^a^	<0.001 (29.389)	0.040 (0.792)	0.010 (0.995)
C20:3n3	0.53±0.03^c^	0.54±0.03^c^	0.57±0.02^c^	0.64±0.05^b^	0.68±0.04^a^	<0.001 (16.194)	0.039 (0.868)	0.026 (0.908)
C20:5n3(EPA)	4.40±0.04^d^	4.51±0.03^c^	4.57±0.04^c^	4.73±0.04^b^	4.85±0.05^a^	<0.001 (103.556)	0.027 (0.849)	0.026 (0.853)
C22:5n3(DPA)	0.36±0.03^c^	0.41±0.04^b^	0.42±0.03^b^	0.51±0.03^a^	0.54±0.04^a^	<0.001 (27.200)	0.030 (0.928)	0.033 (0.902)
C22:6n3(DHA)	3.52±0.08^e^	3.65±0.04^d^	3.76±0.06^c^	3.86±0.05^b^	4.11±0.08^a^	<0.001 (350.736)	0.009 (0.914)	0.003 (0.958)
ΣPUFA	31.76±0.17^e^	32.08±0.14^d^	32.78±0.10^c^	33.80±0.18^b^	34.61±0.12^a^	<0.001 (917.419)	0.024 (0.853)	0.028 (0.837)

*Note*: SFA = Saturated fatty acid; MUFA = Monounsaturated fatty acid; EPA = Eicosapentaenoic acid; DPA = Docosapentaenoic acid; DHA = Docosahexaenoic acid; PUFA = Polyunsaturated fatty acid.

### 3.12 Gene expression

**Fig. 4:**
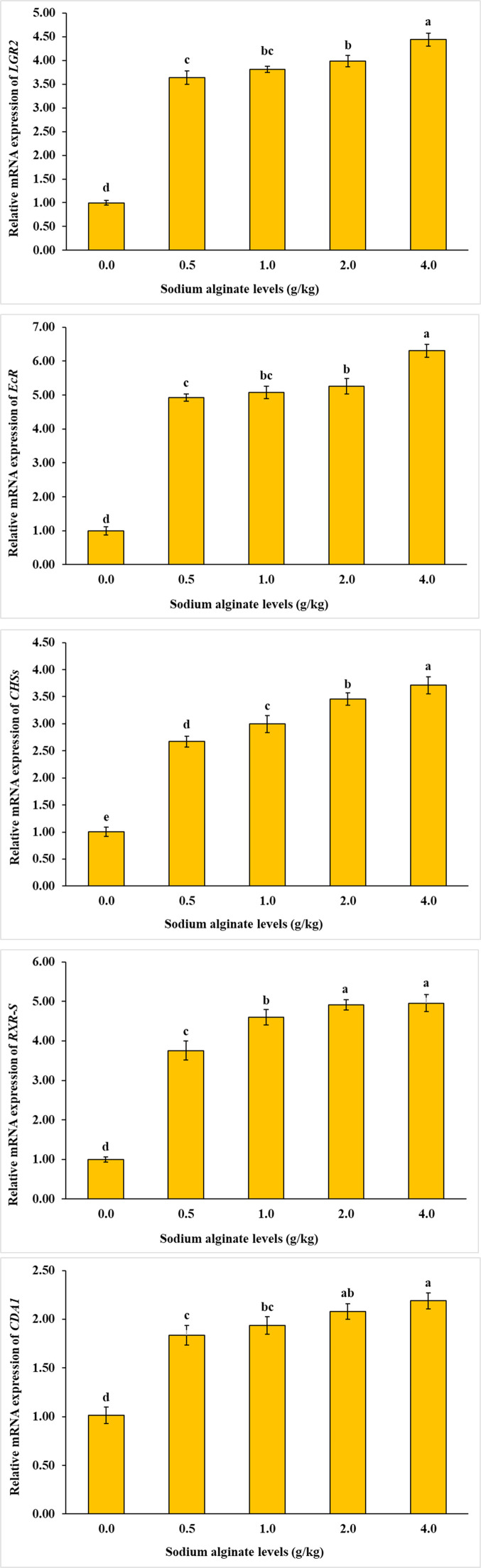
Influence of dietary sodium alginate on *Macrobrachium nipponense*: an assessment of growth-related gene expression over a 60-day feeding period. Values represent mean ± standard deviation (*M* ± SD, *n* = 3). Statistically significant differences among groups are indicated by distinct superscript letters above each bar (*P* < 0.05).

**Fig. 5:**
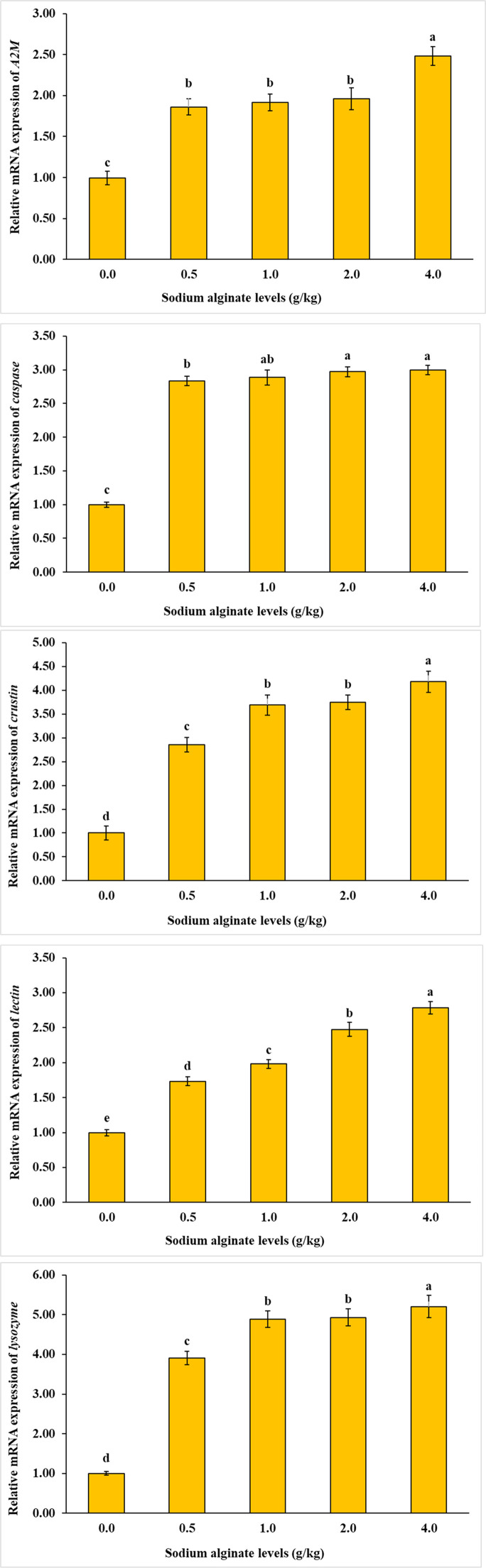
Influence of dietary sodium alginate on *Macrobrachium nipponense*: an assessment of immune-related gene expression over a 60-day feeding period. Values represent mean ± standard deviation (*M* ± SD, *n* = 3). Statistically significant differences among groups are indicated by distinct superscript letters above each bar (*P* < 0.05).

**Fig. 6:**
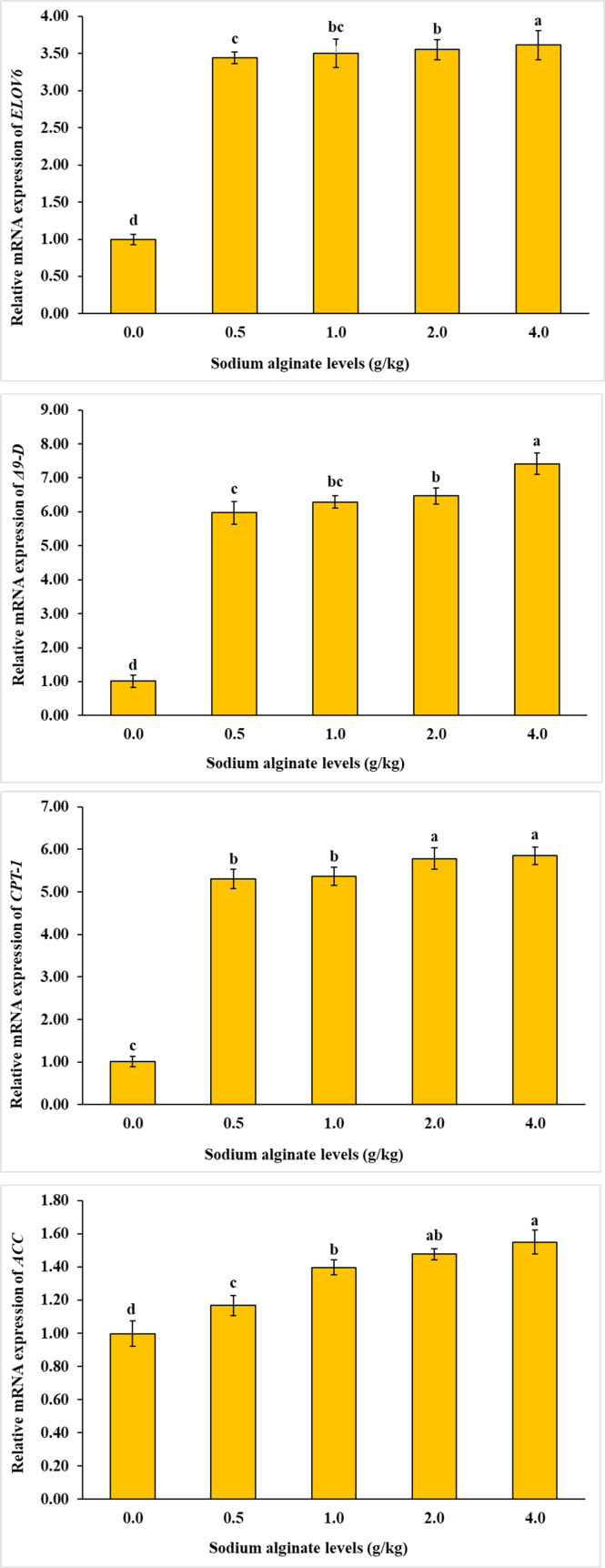
Influence of dietary sodium alginate on *Macrobrachium nipponense*: an assessment of metabolism-related gene expression over a 60-day feeding period. Values represent mean ± standard deviation (*M* ± SD, *n* = 3). Statistically significant differences among groups are indicated by distinct superscript letters above each bar (*P* < 0.05).

As illustrated in Fig. 4, Fig. 5, Fig. 6, the mRNA expression levels of all investigated genes—including *CPT-1, ELOV6, LGR2, CHSs, ACC, CDA1, lectin, A2M, lysozyme, Δ9-D, crustin, RXR-S, caspase*, and *EcR—*were significantly influenced by dietary sodium alginate supplementation. The highest gene expression was detected in the groups receiving 2.0 and 4.0 g/kg of sodium alginate, whereas the control group exhibited the lowest expression levels (*P* < 0.05).

## 4 Discussion

### 4.1 Water quality parameters with emphasis on dissolved oxygen dynamics

Water quality evaluation in this study demonstrated that the majority of physicochemical indices were maintained consistently across all dietary treatments, suggesting that sodium alginate supplementation did not induce detrimental effects on environmental conditions. However, a statistically significant improvement in DO levels was noted in the groups receiving sodium alginate-enriched diets. These findings are supported by previous investigations. For example, Liu et al. (2006) conducted a five-month feeding experiment on black tiger prawn (*Penaeus monodon*), where shrimp were fed diets containing 0, 1, and 2 g/kg of sodium alginate. Their results showed that DO levels in the culture water improved significantly in sodium alginate-fed groups due to enhanced oxygen utilization and reduced oxidative stress in the shrimp, which minimized excessive oxygen consumption and metabolic waste accumulation. Additionally, they observed improved water clarity and reduced nitrogenous waste concentrations. These effects were attributed to the binding capacity of sodium alginate, which can interact with ammonia and nitrite compounds, leading to improved water parameters and healthier aquatic conditions. Oxidative stress arising from inadequate nutrition or environmental fluctuations, can elevate the production of reactive oxygen species (ROS), leading to impaired physiological performance and immune function in aquatic animals (Cheng et al., 2005; Liu et al., 2006; Bai et al., 2023). Sodium alginate, with its inherent antioxidant potential and free radical scavenging ability, mitigates oxidative damage and reduces cellular oxygen demand, thereby contributing to improved DO concentrations in rearing systems (Liu et al., 2006; Yudiati et al., 2016; Zhu et al., 2025). Furthermore, it may support mitochondrial efficiency and stabilize hemolymph oxygen transport by maintaining redox balance (Liu et al., 2006; Jiang et al., 2017; Zhu et al., 2025). Additionally, sodium alginate exhibits effective chelating capacity through its carboxyl and hydroxyl functional groups, enabling it to bind nitrogenous metabolites such as ammonia and nitrite, and reduce their bioavailability in the water column. This process prevents the deterioration of water quality often associated with nitrogenous buildup in high-density aquaculture (Liu et al., 2006; Bai et al., 2023; Wang et al., 2025; Zhu et al., 2025).

The improvement in DO levels observed in this study is particularly noteworthy, as it represents one of the most promising outcomes of sodium alginate supplementation. Dissolved oxygen is a critical water quality parameter influencing respiration, metabolism, and immune competence in crustaceans. Even subtle increases in DO can enhance aerobic efficiency, reduce physiological stress, and improve overall survival (Cheng et al., 2005; Liu et al., 2006; Bai et al., 2023). Our findings indicate that sodium alginate not only benefits the host directly through antioxidant and immunomodulatory mechanisms but also indirectly contributes to a healthier rearing environment by elevating DO levels (Liu et al., 2006; Yudiati et al., 2016). This dual mode of action highlights sodium alginate as a functional additive capable of simultaneously improving animal performance and aquaculture system stability. Such effects have profound implications for intensive farming, where maintaining optimal water quality is often a limiting factor (Liu et al., 2006; Yudiati et al., 2016; Wang et al., 2025; Zhu et al., 2025). These combined effects of sodium alginate—ranging from oxygen conservation and ROS control to nitrogenous waste reduction—underscore its utility as a dietary additive that contributes to maintaining a stable and health-promoting aquatic environment.

### 4.2 Growth indices

The supplementation of sodium alginate in the experimental diets significantly enhanced growth indices, including WG and SGR, while simultaneously reducing the FCR. These observations are in agreement with findings reported by Santos et al. (2019) and Bagheri et al. (2023), who noted that sodium alginate improved growth efficiency and nutrient utilization in L. *vannamei*. Sodium alginate acts as a dietary prebiotic, promoting the proliferation of beneficial gut microbiota and suppressing pathogenic bacteria, which supports both digestive function and immune protection (Cheng et al., 2005; Adilah et al., 2022; Wang et al., 2025). As a fermentable fiber, sodium alginate stimulates short-chain fatty acid (SCFA) production and promotes mucosal health, enhancing the secretion of digestive enzymes such as amylase and protease (Hindu et al., 2018; Niculescu & Grumezescu, 2022; Zhu et al., 2025). It also improves the structural morphology of the gut, particularly by increasing villus length and surface area for absorption. These adaptations contribute to improved digestibility of macronutrients, particularly lipids and proteins, thereby optimizing feed efficiency (Cheng et al., 2005; Adilah et al., 2022; Wang et al., 2025). Sodium alginate enhances digestive enzyme activity and improves intestinal villus morphology, likely contributing to increased growth (Hindu et al., 2018; Niculescu & Grumezescu, 2022). Furthermore, it may influence molting physiology by modulating chitinase activity, thereby reducing molting intervals and accelerating exoskeleton development, both of which are critical for the growth cycles of crustaceans (Immanuel et al., 2012; Santos et al., 2019; Bagheri et al., 2023). Hindu et al. (2018) and Adilah et al. (2022) reviewed several studies on crustaceans and reported that sodium alginate supplementation not only stimulates digestive enzyme production but also acts as a prebiotic to enhance beneficial gut microbiota. These microbial populations are known to secrete exogenous enzymes and vitamins, further contributing to nutrient breakdown and growth promotion. Although mortality was negligible in the present study, as indicated by SR close to 100 % in sodium alginate-supplemented groups, it is worth noting that mortality is often a major challenge in commercial prawn aquaculture. Functional feed additives, including polysaccharides such as sodium alginate, are frequently reported to improve resilience and reduce mortality under stressful farming conditions (Cheng et al., 2005; Immanuel et al., 2012; Bagheri et al., 2023). Therefore, while the present trial did not demonstrate significant mortality, the improvement in survival rate in sodium alginate-fed groups reinforces its potential application as a protective dietary intervention against stress- or pathogen-induced losses in intensive aquaculture systems.

### 4.3 Biochemical parameters

Sodium alginate supplementation significantly altered several hemolymph biochemical markers, excluding calcium and phosphorus. Reductions in glucose, triglycerides, cholesterol, creatinine, urea, and uric acid were observed, while HDL and LDL levels were elevated in treated groups. These findings are consistent with studies by Bagheri et al. (2023), who reported that sodium alginate modulates key metabolic and stress-related indicators in L. *vannamei*. Researchers found that higher doses led to significant reductions in glucose and total cholesterol levels, likely due to enhanced insulin-like peptide activity and improved hepatopancreatic function. In addition, creatinine and urea levels decreased, indicating better excretory function and lower protein catabolism. Under nutritional or environmental stress, nucleic acid catabolism accelerates, leading to the release of nitrogenous wastes such as urea and uric acid. Sodium alginate may mitigate this effect by upregulating uricolytic enzymes, including uricase and allantoinase, which detoxify metabolic byproducts and enhance resilience (Hindu et al., 2018; Wu et al., 2018; Santos et al., 2019; Bagheri et al., 2023; Wang et al., 2025). The observed decrease in creatinine concentrations also implies improved muscular metabolism and excretory efficiency, particularly in the antennal glands responsible for nitrogenous waste excretion (Santos et al., 2019; Bagheri et al., 2023; Wang et al., 2025). In crustaceans, glucose elevation is a hallmark of stress-induced gluconeogenesis, commonly triggered by catecholamine release. Sodium alginate potentially counteracts this by stimulating insulin-like peptide synthesis and promoting glycogen storage in hepatopancreatic tissues, thereby reducing hyperglycemia (Niculescu & Grumezescu, 2022; Bagheri et al., 2023; Zhu et al., 2025). Moreover, its hypolipidemic effects, evidenced by lowered cholesterol and triglyceride levels, suggest enhanced hepatic lipid metabolism and reduced oxidative stress—a common outcome of sodium alginate's antioxidant activity (Liu et al., 2006; Yudiati et al., 2016; Santos et al., 2019; Wang et al., 2025). The rise in HDL and LDL levels, while counterintuitive, may reflect improved lipid transport dynamics and cholesterol redistribution, indicating balanced lipid metabolism rather than pathological dysregulation (Cheng et al., 2004; Wu et al., 2018; Bai et al., 2023).

### 4.4 Non-specific immune responses

The administration of dietary sodium alginate markedly enhanced multiple non-specific immune markers, such as THC, ALB, differential hemocyte counts (HC, GC, and SGC), and TP, while concurrently decreased CORT concentrations. These findings are consistent with previous research by Jiang et al. (2017) and Hindu et al. (2018), who observed that sodium alginate-enhanced diets upregulated immune responses and stabilized hematological indices in L. *vannamei* and *M. rosenbergii*. Sodium alginate’s antioxidant capacity plays a pivotal role in shielding hemocytes from oxidative stress, stimulating cellular proliferation and preserving immune cell function (Wu et al., 2018; Bagheri et al., 2023; Bai et al., 2023; Wang et al., 2025). Wu et al. (2018) investigated the immunomodulatory effects of sodium alginate on the non-specific immune response of the spiny lobster, *Panulirus interruptus*. Their study aimed to evaluate the potential of these compounds to enhance the innate immune system of crustaceans. The results demonstrated that supplementation with sodium alginate led to significant increases in THC, PO activity, and respiratory burst activity. These findings suggest that sodium alginate acts as an effective immunostimulant, enhancing the lobster's innate immune defenses. ALB, synthesized in the hepatopancreas, functions as a marker of protein metabolism and hepatopancreatic health. Its increase in sodium alginate-fed prawns suggests improved hepatic functionality and anabolic status (Yudiati et al., 2019; Bai et al., 2023; Saleh et al., 2025). Similarly, the rise in TP—essential for osmotic balance and immune modulation—further supports alginate’s immunostimulatory effect under stress (Wu et al., 2018; Saleh et al., 2025; Wang et al., 2025). The marked reduction in circulating CORT implies modulation of the neuroendocrine axis and decreased physiological stress, further corroborated by enhanced enzyme-based defenses. Sodium alginate-fed prawns also exhibited significantly increased LYZ and PO activities, reinforcing its ability to stimulate innate immune mechanisms. Yudiati et al. (2016) investigated the immunostimulatory effects of dietary alginate on L. *vannamei* over a 30-day feeding period, using three different alginate types (A1, A2, A3) at an inclusion rate of 1.5 *g*/kg in the diet. The results demonstrated that alginate supplementation significantly enhanced key immune parameters in shrimp. PO activity and respiratory burst were markedly increased in all alginate-fed groups compared to the control. In our study, reduced AST, ALT, and LDH levels further confirmed lower hepatopancreatic damage and stress. Moreover, stable ACP and AKP levels point to preserved membrane stability and lysosomal integrity (Liu et al., 2006; Yudiati et al., 2019; Bai et al., 2023; Saleh et al., 2025; Zhu et al., 2025). Bagheri et al. (2023) investigated the effects of dietary low molecular weight sodium alginate (LMWSA) supplementation on key enzymatic parameters of L. *vannamei* over an 8-week feeding period. They observed that enzymatic indicators—ALT and AST—remained stable in the sodium alginate-supplemented groups, suggesting reduced physiological stress and a positive effect on enzymatic immune parameters. Together, these immune responses reflect enhanced systemic resilience and improved energy allocation toward growth and defense in sodium alginate-supplemented prawns.

The study revealed a marked upregulation of pivotal genes regulating growth, immune responses, and metabolic pathways in prawns receiving sodium alginate-supplemented diets. Similar gene expression patterns have been reported by Yudiati et al. (2019) and Bai et al. (2023) in crustacean models under alginate feeding regimes. Santos et al. (2019) examined the effects of dietary sodium alginate supplementation on the expression of Toll-like receptor (TLR) genes in L. *vannamei* over a 30-day experimental period. The study measured mRNA levels of multiple TLR genes, including TLR3, TLR4, and TLR5, using quantitative PCR to evaluate the immune-modulatory impact of sodium alginate. Results demonstrated a significant upregulation of these TLR genes in shrimp fed diets supplemented with sodium alginate compared to controls. This enhanced gene expression suggests that sodium alginate acts as an immunostimulant by activating key pattern recognition receptors involved in innate immune responses. In a similar molecular investigation, Yudiati et al. (2019) studied the effects of dietary sodium alginate supplementation on immune gene expression and resistance to White Spot Syndrome Virus (WSSV) in L. *vannamei*. The study administered sodium alginate at concentrations of 3.0, 4.5, and 6.0 g/kg of feed for 14 days. Quantitative real-time PCR (qRT-PCR) was employed to assess the expression levels of immune-related genes, including those encoding TLRs and lectins, both before and after WSSV challenge. Results demonstrated that shrimp fed with sodium alginate at 6.0 g/kg exhibited a significant upregulation of TLR and lectin gene expression compared to the control group. Notably, this upregulation was sustained at 48 h’ post-infection, correlating with enhanced resistance to WSSV, as evidenced by a 56 % survival rate in the alginate-treated group versus 10 % in controls. These findings underscore the potential of sodium alginate as an effective immunostimulant, enhancing innate immune responses through the modulation of gene expression, thereby improving disease resistance. Sodium alginate appears to activate pattern recognition receptors such as TLRs, which identify microbial-associated molecules and trigger immune signaling cascades (Yudiati et al., 2016; Bai et al., 2023; Wang et al., 2025; Zhu et al., 2025). This stimulation leads to enhanced expression of immune effector genes and contributes to increased resistance against pathogens. Simultaneously, genes associated with metabolic homeostasis and tissue development are also activated, indicating that sodium alginate may influence endocrine and cellular pathways related to growth. These genetic modulations affirm the multifunctional role of alginate as a dietary bioactive with systemic benefits for crustaceans (Santos et al., 2019; Yudiati et al., 2019; Bai et al., 2023; Wang et al., 2025).

### 4.5 Antioxidant activities

Our findings revealed that dietary sodium alginate markedly decreased MDA, SOD, and CAT levels while enhancing T-AOC. Interestingly, GPx levels remained unchanged across treatments. These results align with previous work by Cheng et al. (2004) and Bai et al. (2023), who reported that sodium alginate improved oxidative stress markers and enhanced antioxidant defense in L. *vannamei*. Jiang et al. (2017) studied the effects of dietary supplementation with 1.0 g/kg alginate-derived oligosaccharides (ADOs) on the immune response of L. *vannamei* over a 30-day experimental period. Their results showed that the alginate-fed group significantly enhanced antioxidant defenses. This boost in antioxidant capacity helped reduce oxidative stress in shrimp, which is crucial for maintaining immune function and improving resistance against infections. The antioxidant capacity of aquatic organisms is influenced by both reactive oxygen species (ROS) load and the efficiency of enzymatic detoxification systems (Cheng et al., 2004; Jiang et al., 2017; Bagheri et al., 2023). Elevated T-AOC levels reflect the organism’s systemic ability to neutralize free radicals and maintain redox equilibrium. Decreased MDA levels in sodium alginate groups suggest reduced lipid peroxidation, while reduced CAT and SOD levels may indicate decreased oxidative pressure, hence lowering the demand for these enzymes (Jiang et al., 2017; Bagheri et al., 2023; Bai et al., 2023). Immanuel et al. (2012) demonstrated that sodium alginate reduces oxidative biomarkers and restores redox homeostasis in black tiger prawn (*P. monodon*) challenged with white spot syndrome virus. Sodium alginate likely scavenges superoxide and hydroxyl radicals directly, thereby reducing oxidative damage and preserving cellular integrity without over-activating detoxification enzymes (Jiang et al., 2017; Bai et al., 2023; Wang et al., 2025). Cheng et al. (2005) evaluated the effects of dietary sodium alginate (0.5, 1.0, and 2.0 g/kg) over five months on the immune and antioxidant responses of juvenile L. *vannamei*. Shrimp fed 2.0 g/kg sodium alginate showed significantly increased activities of SOD and respiratory burst, indicating enhanced ability to neutralize reactive oxygen species. Although GPx activity decreased. The maintenance of improved antioxidant responses and reduced lipid and ROS-associated enzymes, supports the hypothesis that sodium alginate mitigates oxidative stress through both direct and indirect mechanisms (Cheng et al., 2005; Liu et al., 2006; Yudiati et al., 2016; Bagheri et al., 2023). These effects reduce the energy costs associated with defense responses, enabling prawns to reallocate resources toward growth and metabolic efficiency.

### 4.6 Digestive enzyme activity

The present study showed that prawns receiving sodium alginate exhibited a significant increase in the activity of digestive enzymes such as amylase, protease, and lipase. This finding is consistent with prior research by Cheng et al. (2005), Hindu et al. (2018), and Adilah et al. (2022), who observed that alginate supplementation enhances enzymatic secretion and nutrient assimilation in crustaceans. Yarahmadi et al. (2023) investigated the effects of dietary sodium alginate supplementation on digestive enzyme activities in yellowfin sea bream (*Acanthopagrus latus*) over a 56-day experimental period. The study evaluated the activities of key digestive enzymes including trypsin, lipase, and amylase in the intestines of fish fed diets containing varying levels of sodium alginate. Results demonstrated a significant increase in these enzyme activities in fish receiving alginate supplementation compared to the control group, indicating enhanced digestive capacity. The researchers also measured growth parameters and body composition to correlate enzyme activity with nutritional status, providing a comprehensive assessment of sodium alginate’s impact on digestive physiology. Histological evaluation revealed longer intestinal villi and greater goblet cell numbers, indicating improved digestive surface area and mucosal health. Sodium alginate’s prebiotic properties foster the growth of beneficial gastrointestinal bacteria, including *Bacillus* and *Lactobacillus* spp*.*, which secrete complementary exogenous enzymes that synergize with the host’s endogenous enzyme systems (Cheng et al., 2005; Hindu et al., 2018; Adilah et al., 2022; Wang et al., 2025). Additionally, the acidic microenvironment promoted by sodium alginate enhances microbial fermentative activity, leading to improved digestion and nutrient availability (Niculescu & Grumezescu, 2022; Yarahmadi et al., 2023; Zhu et al., 2025). These results collectively indicate that sodium alginate enhances the digestive capacity of prawns through both direct modulation of gut physiology and indirect support via microbial interactions.

### 4.7 Intestinal microflora

Dietary sodium alginate markedly decreased TBC while promoting the proliferation of LAB in the intestinal tract. These results corroborate the findings of Cheng et al. (2005), Hindu et al. (2018), and Adilah et al. (2022), who reported that sodium alginate modulates intestinal microflora composition in favor of beneficial bacterial species. Asaduzzaman et al. (2019) investigated the effects of dietary sodium alginate on gut microbiota composition in Malaysian Mahseer, *Tor tambroides* over a 60-day experimental period. Juvenile fish were fed diets supplemented with sodium alginate at levels of 0 %, 0.1 %, 0.2 %, 0.4 %, and 0.8 %. High-throughput sequencing revealed that supplementation with ≥0.2 % sodium alginate led to significant shifts in gut microbial communities. Notably, there was an increase in beneficial bacterial groups. In particular, genera such as *Lactobacillus, Bifidobacterium*, and *Bacillus* are among the most commonly reported beneficial bacteria stimulated by sodium alginate supplementation in aquatic species (Cheng et al., 2005; Wang et al., 2025). These bacteria not only enhance digestive enzyme secretion but also contribute to competitive exclusion of pathogens (Hindu et al., 2018; Adilah et al., 2022). Considering this, it is reasonable to hypothesize that sodium alginate could be strategically combined with such probiotics in the form of synbiotics (Cheng et al., 2005; Hindu et al., 2018; Adilah et al., 2022; Zhu et al., 2025). Although synbiotic application was not directly tested in the present study, this approach may provide synergistic benefits by simultaneously promoting beneficial bacterial colonization and supplying a selective substrate, thereby further enhancing gut health, immunity, and growth performance in prawns.The ability of sodium alginate to lower intestinal pH (to 5.0–6.5) and maintain anaerobic, CO₂-enriched microenvironments suppresses pathogenic gram-negative bacteria and the growth of acidophilic, Gram-positive LAB including *Lactobacillus* spp. (Niculescu & Grumezescu, 2022; Yarahmadi et al., 2023; Zhu et al., 2025). This prebiotic effect reinforces gut health and contributes to digestive efficiency. Furthermore, the enhancement of LAB populations directly supports improved digestive enzyme activity and nutrient assimilation, forming a synergistic feedback loop that optimizes host growth and immune function (Cheng et al., 2005; Hindu et al., 2018; Adilah et al., 2022; Wang et al., 2025).

### 4.8 Body composition profiles

Sodium alginate inclusion was positively associated with increased whole-body protein and lipid levels, accompanied by a decrease in moisture and ash content. The researchers attributed these improvements to enhanced nutrient absorption, improved antioxidant defense, and reduced oxidative degradation of muscle tissue. The enhanced protein accumulation is likely due to alginate’s role in stabilizing amino acid metabolism and promoting transamination reactions in muscle tissue (Yudiati et al., 2016; Niculescu & Grumezescu, 2022; Yarahmadi et al., 2023; Wang et al., 2025; Zhu et al., 2025). Additionally, its antioxidant activity reduces lipid peroxidation and promotes lipid retention, resulting in higher mono- and polyunsaturated fatty acid (MUFA, PUFA) content and reduced saturated fatty acid (SFA) levels (Bai et al., 2023; Yarahmadi et al., 2023; Wang et al., 2025). These compositional improvements are essential not only for animal growth but also for product quality in aquaculture. Sodium alginate facilitates amino acid preservation in muscle tissue by enhancing nitrogen retention through reduced catabolism. Its bioadhesive and antioxidant properties also help maintain membrane integrity, contributing to higher lipid and protein stability (Cheng et al., 2005; Jiang et al., 2017; Bai et al., 2023; Yarahmadi et al., 2023). These results collectively suggest that sodium alginate improves the biochemical quality and nutritional profile of prawn tissue by promoting anabolic activity, preserving fatty acid integrity, and minimizing cellular dehydration.

## 5 Conclusion

Our study provides compelling evidence that dietary sodium alginate exerts multifaceted benefits on *Macrobrachium nipponense*, significantly enhancing growth performance, digestive enzyme activity, immune responses, and antioxidant status. The observed improvements in physiological and biochemical parameters affirm the functional role of sodium alginate as an effective prebiotic and immunostimulant in crustacean nutrition. Notably, dietary supplementation at a level of 4.0 g/kg resulted in optimal biological outcomes, particularly in growth indices and immune modulation. The integration of sodium alginate into aquafeeds appears to support nutrient assimilation, promote gut microbial equilibrium, and alleviate oxidative stress—mechanisms that collectively contribute to better health and performance in farmed freshwater prawns. These findings not only reinforce the potential of alginate as a sustainable and natural alternative to synthetic additives, but also lay the foundation for precision diet formulation tailored to species-specific physiological demands. Nevertheless, while the results of the current study highlight a promising dietary strategy, they also underscore the need for further investigation. Future studies should evaluate long-term effects of alginate supplementation across diverse developmental stages, environmental conditions, and health statuses. In particular, dose-response trials stratified by body weight classes and stressor challenges will be instrumental in refining optimal inclusion levels for commercial applications. A deeper exploration of gene expression dynamics and microbiome modulation will also expand our understanding of sodium alginate's systemic influence in crustaceans.

## Ethics statement

All methodologies involving animal subjects were executed in accordance with the ethical standards established by the institution and complied with the guidelines stipulated in Directive 2010/63/EU. All pertinent experimental protocols conformed to the recognized principles and regulations governing the management of issues pertaining to experimental animals.

## Data availability

Data will be made available on request.

## Funding

This research received no specific grant from any funding agency in the public, commercial, or not-for-profit sectors.

## CRediT authorship contribution statement

**Mohammad Ettefaghdoost:** Writing – review & editing, Writing – original draft, Visualization, Supervision, Resources, Project administration, Methodology, Investigation, Funding acquisition, Formal analysis, Data curation, Conceptualization. **Hossein Haghighi:** Writing – review & editing, Validation, Software, Methodology, Investigation, Formal analysis.

## Declaration of competing interest

The authors declare that they have no known competing financial interests or personal relationships that could have appeared to influence the work reported in this paper.
